# Effects of Two Early Parenting Programmes on Child Aggression and Risk for Violence in Brazil: a Randomised Controlled Trial

**DOI:** 10.1007/s11121-024-01698-3

**Published:** 2024-07-02

**Authors:** Joseph Murray, Rafaela Costa Martins, Melanie Greenland, Suélen Cruz, Elisa Altafim, Adriane Xavier Arteche, Peter J. Cooper, Marlos Rodrigues Domingues, Andrea Gonzalez, Adriana Kramer Fiala Machado, Lynne Murray, Isabel Oliveira, Iná Santos, Tâmara Biolo Soares, Luciana Tovo-Rodrigues, Merryn Voysey

**Affiliations:** 1 Human Development and Violence Research Centre, Federal University of Pelotas, Pelotas, Brazil; 2 Postgraduate Program in Epidemiology, Federal University of Pelotas, Pelotas, RS Brazil; 3 Oxford Vaccine Group, Department of Paediatrics, University of Oxford, Oxford, UK; 4 Mental Health Postgraduate Program, Ribeirão Preto Medical School, University of São Paulo, São Paulo, Brazil; 5 Postgraduate Program in Psychology, Pontifícia Universidade Católica Do Rio Grande Do Sul (PUCRS), Porto Alegre, Brazil; 6 School of Psychology and Clinical Language Sciences, University of Reading, Reading, UK; 7 Postgraduate Program in Physical Education, Federal University of Pelotas, Pelotas, Brazil; 8 Department of Psychiatry & Behavioural Neurosciences, McMaster University, Hamilton, Canada; 9 Instituto Cidade Segura, Porto Alegre, Brazil

**Keywords:** Parenting programmes, Aggression, Violence, Early childhood, Randomised control trial

## Abstract

Violence is a major public health problem globally, with the highest rates in low- and middle-income countries (LMICs) in the Americas and southern Africa. Parenting programmes in high-income countries can diminish risk for violence, by reducing risk factors such as child aggression and harsh parenting, and increasing protective factors such as child cognitive development and school readiness. However, there is critical need to identify low-cost programmes with replicable benefits that work in real-world LMICs contexts. A three-arm, randomised, single-blind trial evaluated effects of two low-cost, group-based parenting programmes recommended for LMICs (ACT: Raising Safe Kids; DBS: dialogic book-sharing) on child aggression (primary outcome), child development, parenting, maltreatment, and stress. Participants were 369 children with medium–high levels of aggression (mean age 3.1 years at baseline) in poor households. Interventions were implemented in city health and education services in southern Brazil. Maternal reports, filmed observations, child tasks, and hair cortisol were assessed at baseline, 1-month post-intervention, and 8-month follow-up. Intention-to-treat analyses compared each of ACT and DBS with a control group. Three hundred sixty-eight (99.7%) participants completed follow-up assessments 8 months after the interventions. There was no effect of ACT (standardised mean difference, SMD 0.11, 95% CI − 0.05, 0.27) or DBS (SMD 0.05, 95% CI − 0.11, 0.21) on the primary outcome of child aggression. ACT reduced harsh parenting behaviour post-intervention (SMD − 0.23; 95% CI − 0.46, − 0.01), but not at follow-up. DBS improved book-sharing practices at both time points (e.g., maternal sensitivity at follow-up SMD 0.33; 95% CI 0.08, 0.57). There were no benefits of either programme for other parenting, child development, or stress outcomes. Two parenting programmes in Brazil had small effects on parenting practices but did not reduce child aggression or several other important risk/protective factors for violence. Effective early interventions that reduce violence in real-world LMIC settings are highly desirable but may be challenging to achieve.

**Supplementary Information:**

The online version contains supplementary material available at 10.1007/s11121-024-01698-3.

## Introduction

An estimated 300 million (3 in 4) children 2–4 years old experience violent discipline by caregivers each month (UNICEF, 2017), and many more suffer exposure to other forms of violence, both in the home and community. Interpersonal violence has major social, health, and economic costs in low- and middle-income countries (LMICs), particularly in the Americas and sub-Saharan Africa where the highest rates of severe violence are found (Institute for Health Metrics and Evaluation, 2022). Decades of longitudinal research in both high-income and LMICs shows that exposure to violence in childhood and persistent child aggression are key predictors of later perpetration of violence (Broidy et al., 2003; Murray et al., 2018), suggesting the importance of early prevention. A growing evidence base in LMICs demonstrates that parent-training programmes have substantial benefits for child development (Jeong et al., 2021), and potentially such programmes could also prevent violence by reducing harsh parenting, as well as children’s own aggression before it becomes entrenched.

Currently, the strongest evidence that early interventions can prevent violence comes from evaluations of typically intensive and lengthy parenting programmes in North America and other high-income countries (Farrington & Welsh, 2007), but these are often commercialised with unrealistic costs for LMICs (Mejia et al., 2017). Recognising the need for new, low-cost programmes in the Global South, the World Health Organization (World Health Organization, 2022) is promoting evaluation and dissemination of free parent-training programmes with low implementation costs, including two group-based parenting programmes, which we evaluate in the current study: a dialogic book-sharing programme (DBS) and a programme aiming to reduce harsh parenting (ACT: Raising Safe Kids). Group-based programmes are particularly relevant to consider for scale-up in LMICs because they have lower costs than individualised interventions and can be just as effective (Luoto et al., 2021).

The group-based DBS programme we evaluate is included in the WHO Parenting for Lifelong Health Package (World Health Organization, 2022) and had previously been found to improve positive parenting and benefit child language and attention (Murray et al., 2016a, b; Vally et al., 2015). It also aims to increase child socio-emotional understanding. Because these are important protective factors against the development of aggression (Eisner & Malti, 2015; Murray et al., 2018), DBS could potentially reduce child aggression — and risk for later violence.

The ACT programme (American Psychological Association, 2022) we evaluate aims to support carers to raise children free of violence — a critical outcome in itself, and also another major influence on children’s own aggressive behaviours (Gershoff, 2002). Thus, DBS and ACT each target different types of parenting practices and child factors known to influence aggression and risk for violence, both by and against children. The appeal of such low-cost programmes is strong: if they can successfully reduce risk for violence early in life, as well as support child development, they would be attractive policy options in low-resource settings, where violence imposes large burdens for health and society.

At the time of the current trial, evidence on the effectiveness of parenting programmes to reduce aggression and violence in LMICs was limited. A rare and important Jamaican study had shown that a 2-year stimulation programme provided to poorly nourished children in the 1980s reduced violence 20 years later (Walker et al., 2011). A meta-analysis of another eight trials of parenting programmes in LMICs found a significant average reduction in child conduct problems, but there was considerable heterogeneity in results (Burkey et al., 2018). Several further individual trials have also reported positive results (e.g., Altafim & Linhares, 2019; Cova et al., 2019; Jensen et al., 2021; Lachman et al., 2021; Ward et al., 2020), but one found no main effects on child behaviour, despite improvements in parenting (Francis & Baker-Henningham, 2021). The latest, large, systematic review of parenting programmes for children aged 2–17 in LMICs found moderate evidence for short-term effectiveness, but considerable heterogeneity in results, and common methodological weaknesses (Backhaus et al., 2023). Thus, it remains unclear which programmes have replicable effects and should be implemented as public policy in LMICs. Because many programmes have been delivered by research staff or other specialised personnel, a top priority is to identify those that achieve effects in real-world LMIC settings for scalable implementation (Tomlinson et al., 2019).

To our knowledge, only one LMIC parenting trial to date has specifically evaluated child conduct problems as a pre-registered primary outcome, finding no effects in a sample of 68 low-income families in South Africa (Lachman et al., 2017). Another limitation in the evidence base is that, with some exceptions (e.g., Dowdall et al., 2021; Lachman et al., 2017; Ward et al., 2020), most LMIC trials have measured outcomes using only self-reports, and normally only immediately post-intervention. Reviews also note that many LMIC trials have additional problems such as no pre-registration, non-blinded coding of outcomes, or considerable attrition (Backhaus et al., 2023; Burkey et al., 2018; Knerr et al., 2013; Pedersen et al., 2019). Hence, new, high-quality evaluations of low-cost programmes relevant to public policy in LMICs are critically needed. We evaluated the effects of ACT and DBS in Pelotas, Brazil where rates of violence are high (Murray et al., 2015), and the local government aimed to scale up these programmes as public policy.

## Methods

A three-arm, single-blind, randomised controlled trial, named the Pelotas Trial of Parenting Interventions for Aggression (PIÁ; *Primeira Infância Acolhida*), was conducted to test the effects of the ACT and DBS parenting programmes on a primary outcome of child aggression, and secondary outcomes of child development, parenting, maltreatment, and stress. Participants were allocated 1:1:1 to ACT, DBS, or Control (receiving services as usual). The trial was pre-registered (Brazilian Ministry of Health Register of Clinical Trials: RBR-2kwfsk), a protocol was published before the end of recruitment (Murray et al., 2019), and a statistical analysis plan was pre-specified (Supplement, Appendix B).

### Participants, Eligibility, and Recruitment

Participants were first selected from the 2015 Pelotas Birth Cohort Study (Hallal et al., 2018), which is a total population-based, prospective cohort study, including all children born between January and December 2015 in Pelotas, a city of approximately 340,000 people in Southern Brazil. The cohort consists of 4275 children — 98.7% of all children live-born in the city that year. Based on a follow-up assessment of 4078 cohort children at age 24 months, families potentially eligible for the PIÁ trial were identified as follows: (i) residence geocoded in Pelotas, (ii) family income in the bottom 30% of the cohort, (iii) biological mother cared for child, (iv) mothers and children without visual, speech or auditory impairment, (v) singleton child (not multiple birth), (vi) child did not screen for severe developmental delay (in lowest 10% on INTER-NDA; see Supplement Table S7), and (vii) child scored above the bottom third of aggressive behaviour ratings on the ELDEQ (Étude Longitudinale du Développement des Enfants du Québec) study questionnaire (see Supplement Table S3). When children were aged mean 3.1 years (PIÁ trial baseline), the 752 potentially eligible families were divided into 11 geographic areas in Pelotas city and were visited to confirm eligibility and invite mothers to participate in the trial, until the required sample size was achieved.

Of the 752 families identified as potentially eligible for the trial based on age 24-month cohort data, 383 were not included in the trial because they had subsequently moved or were unable to participate for other practical reasons (*n* = 84), declined to participate (*n* = 96), received both ACT and DBS (*n* = 31),^1^ or were not contacted because the trial sample size was already achieved (*n* = 170). Thus, the final trial sample consisted of 369 families.

### Interventions

Two, brief, group-based, manualised parenting programmes (ACT and DBS), were delivered between July and December 2018, in public educational facilities. Details about the content of each intervention are in the Supplement, and a brief description is provided below. Given their low-resource requirements, ACT and DBS are potentially scalable for low-resource settings and are promoted in the influential WHO-led INSPIRE framework for violence prevention (World Health Organization, 2016). Programme implementation was done in collaboration with the Pelotas municipal government, which aimed to make ACT and DBS public policy, while assessments were conducted entirely independently by the research team. The control group continued to receive services as usual in the community.

#### Intervention 1: ACT

The ACT Raising Safe Kids Program was developed by the American Psychological Association as a non-profit, low-cost intervention (American Psychological Association, 2022). It consists of nine group-based, two-hour, weekly sessions, in which parents are trained without children present (through interactive activities, slides, and videos) about child development, strategies for emotion and behaviour regulation, positive communication, problem-solving techniques, and guidance on how to raise children free of violence. ACT has been implemented in 14 countries, and at the time of the current trial had been previously adopted for use in Brazil by the programme developer and the senior psychologist who trained facilitators in Pelotas. It is now being implemented in over 20 municipalities in Brazil.

#### Intervention 2: DBS

The WHO Violence and Injuries Prevention Unit has assembled a suite of free parenting interventions (Parenting for Lifelong Health) aimed at reducing risk factors and increasing protective factors against violence in LMICs (Ward et al., 2016; World Health Organization, 2022). One of these is a dialogic book-sharing programme developed by the Mikhulu Trust. Central features of DBS are that, while sharing a picture book with a child, the parent follows the child’s focus of interest, responds sensitively, and engages the child in reciprocal exchanges about the book content, including talking about the emotions and perspectives of the book characters. Training is delivered weekly over eight weeks to small groups of parents in 90-min sessions, with parents practicing with their children and receiving feedback, and books provided for practice at home. The programme is easily culturally transportable and has been implemented in 11 countries. Programme developers supervised translation and adaptation of content and ran workshops in Pelotas to train local facilitators for this trial. With expansion of DBS to other municipalities, by 2022 it was implemented with over five thousand children in Brazil.

ACT and DBS were implemented by facilitators employed by the Pelotas city government health and education sectors, under the supervision of the research team to maximise fidelity and implementation quality. ACT was delivered by pairs of facilitators with a minimum of a graduate degree in a relevant area, nearly all working in municipal schools as education coordinators. DBS was delivered by facilitators of PIM, a health sector home-based programme for child development. Facilitators were trained and certified in ACT (*n* = 13) and DBS (*n* = 12) by master trainers, and received weekly supervision from a senior psychologist for ACT, and from a master trainer for DBS. A random session delivered by each facilitator was rated for fidelity by the research staff. “Good facilitators” were defined as those who covered at least 75% of all content defined in the manual for that session.

Adherence was defined by programme developers as completing at least seven ACT sessions and six DBS sessions. To encourage attendance, mothers were shown videos of previous participants who had positive experiences of the programmes (during piloting of the programmes in Pelotas); sessions were organised around mothers’ agendas, and alternative groups were available when sessions were missed; transport was provided for the first session and on rainy days, and provision was made for childcare, snacks, and costs for transport to the sessions; reminders were sent before sessions, and certificates provided at the end of the course (Martins et al., 2020).

### Randomisation, Masking, and Contamination

Immediately after baseline assessment, trial managers randomised participants to ACT/DBS/Control, minimising (MINIM: Evans et al., 2022) for child age, sex, and aggression, and level of harsh parenting (all binary variables measured at 24 months). Given the nature of the interventions, participants and facilitators were not blinded to intervention. All outcomes were assessed by researchers blind to intervention status, with participants requested not to reveal their status. Coding of filmed observational data was conducted by blinded raters. Programme session registries were examined to identify whether there was any cross-over between each arm, and mothers were asked post-intervention whether they had learned about any interventions that they had not participated in.

### Assessments

A detailed set of pre-specified outcomes was assessed at baseline, 1-month post-intervention, and at 8-month follow-up in maternal interviews, filmed mother–child interactions, child tests, and from biological samples. Measures are summarised below, and details (including references for measures) are in the Supplement (Tables S3–S6). It was important to consider a broad range of possible secondary outcomes of the programmes (as well as effects on the primary outcome of child aggression), given the very limited evidence on these or similar interventions in Brazil, and because the complex nature of the interventions imply many potential benefits (and have been demonstrated for some parenting programmes in other contexts). All outcomes were assessed for both interventions, although some outcome domains were hypothesised to be influenced primarily by one of the programmes (e.g. general child development by DBS; Murray et al., 2019). Assessments were conducted by trained interviewers at the university research centre, lasting approximately one hour. Mothers and children were evaluated separately after direct assessments of mother–child interactions, and children had breaks to maintain engagement. Many assessments took the form of games and puzzles and included toy play or book-sharing with the parent. Maternal questionnaires were read aloud to mothers given literacy issues. Filmed, observational measures were coded by a central team of psychologists, with continual supervision and 10% double-coded for calculation of inter-rater reliability.

#### Child Aggression at Follow-up (Primary Outcome)

Following recommendations of an international, independent trial steering committee, the primary outcome of child aggression was specified as a combination of the aggression sub-scales of the Child Behavior Checklist (CBCL) and the ELDEQ questionnaire (mean of the two z-scores). As secondary outcomes, each questionnaire score was analysed separately, and to minimise bias from using a single source, three observational assessments of child aggression were examined (see Supplement, Table S3).

#### Child Development at Follow-up

We aimed to assess a range of child development factors known to influence trajectories of aggressive behaviour. It was hypothesised that DBS would mainly influence child capacities in language and other skills (Murray et al., 2019). Child language was assessed using the Brazilian Test of Receptive and Expressive Vocabulary. However, at baseline, a quarter of these data were unavailable because of a technical problem, so language data from the most recent cohort assessment (using the INTER-NDA assessment at age 24 months; Fernandes et al., 2020), was included to impute those missing data. Child attention and executive functions were assessed using a parental questionnaire, child tests, and an interviewer rating (Supplement, Table S4), as were child empathy and prosocial behaviour. Child tests were used to evaluate emotion recognition and altruism (Supplement, Table S4); child theory of mind was measured using the triangle task at baseline and, given problems with its implementation, the Sally-Anne task at follow-up.

#### Parenting and Maltreatment at Post-intervention and Follow-up

We aimed to assess multiple dimensions of parenting, both by self-report and observation. Positive parenting and harsh parenting (hypothesised to relate principally to ACT; Murray et al., 2019) were assessed using self-report questionnaires, filmed parent–child interactions during structured and free play tasks, and during book-sharing (Supplement, Table S5). Maltreatment was measured using the Juvenile Victimization Questionnaire (JVQ), asking about any form of probable maltreatment suffered by the child by anyone prior to baseline, and then in the post-intervention period (Supplement, Table S5). Potential cases were referred to senior psychologists who responded appropriately.

#### Stress at Follow-up

It was hypothesised that parenting and child stress might reduce given the support provided by the interventions (Murray et al., 2019). Maternal perceived stress was measured using two questionnaires, and biological stress was measured as 3-month average cortisol concentrations from hair samples of both mothers and children (Supplement Table S6, and Supplement text for details of cortisol processing procedures).

##### Changes to Protocol Outcomes

The pre-specified analysis plan (Supplement, Appendix B) details changes made after the protocol was published, including (i) the addition of a measure of child prosocial behaviour (on the SDQ) and attention (on the Filmed Play Alone Task); (ii) analysis of all parenting outcomes at both post-intervention and follow-up; (iii) the exclusion of official records as a measure of maltreatment given their unavailability.

### Statistical Analysis

The pre-specified analysis plan is available in the Supplement (Appendix B).

The sample size was initially calculated to be able to detect a standardised mean difference (SMD) of 0.45 between interventions and control for the primary outcome with 80% power (similar to effects of well-established programmes reviewed by Piquero et al. (2016) and parent-focussed programmes in LMICs reviewed by Burkey et al. (2018), regarding child antisocial behaviour). For this power, the trial would need 104 participants per group with alpha set at 0.025 (given comparisons between ACT and Control, and between DBS and Control), after adjusting for possible attrition of 10% at follow-up. To allow for identification of potentially smaller effects, and effects at later ages, a larger sample of 369 (*n* > 120 per group) was recruited and there was almost no attrition (see “Results”), leading to increased power in the current analyses.

Analyses were conducted using the intention-to-treat (ITT) population which included all participants who were enrolled and randomised. Planned sensitivity analyses were conducted using the per-protocol (PP) population which included participants who completed the intervention if randomised to an intervention group. All participants in the control arm were included in PP analyses.

The primary outcome and its individual components were first summarised using unadjusted means by allocation and time point. For the main effect estimates, we focus on effect sizes rather than statistical significance (as recommended by Sullivan and Feinn (2012). For continuous outcomes, adjusted SMDs are presented, with 95% confidence intervals (CIs). Binary outcomes were modelled using logistic regression and presented as frequencies, percentages, adjusted odds ratios (ORs), and 95% CIs. Models were adjusted for baseline score, child’s age and sex, maternal education and depression, and outcome-specific covariates (See Supplement Appendix B, Table 2). Within-subject repeated-measures accounted for outcomes that were collected at multiple time points. Mixed effects models with neighbourhood as a random effect were fitted. If models did not converge, neighbourhood was fitted as a fixed effect. Adjustments were not made for multiple comparisons because the outcomes were associated with each other and adjustment would have over-corrected (Schulz & Grimes, 2005). However, given the many outcomes, results were interpreted cautiously, and we focus on findings regarding the primary outcome and those that have consistency of results across different measures in a single domain and clinical plausibility.

Analyses were conducted on a complete case analysis basis except for models where baseline child language was a covariate. Multiple imputation (MI) by chained equations was implemented to account for systematic missing baseline child language in select neighbourhoods. Imputation models included model-specific covariates and language measures collected at the age of 24 months. Pooled estimates are presented for analyses where MI was implemented.

Three sensitivity analyses were conducted. First, for child language outcomes, complete case analyses were conducted. Second, child aggression outcomes were analysed by adjusting for generic covariates only (not also outcome-specific covariates, given some had missing data). Third, for ACT, effects on parenting were estimated for the sample of participants who completed the programme and did so with a high-fidelity facilitator.

Moderator analyses were performed on pre-specified outcome-covariate relationships, and *p*-values are presented for each intervention versus control separately and determine the significance of a categorical term in the statistical model. Mediator analyses were not conducted due to lack of effects on the primary outcome, as per the statistical analysis plan.

## Results

**Fig. 1:**
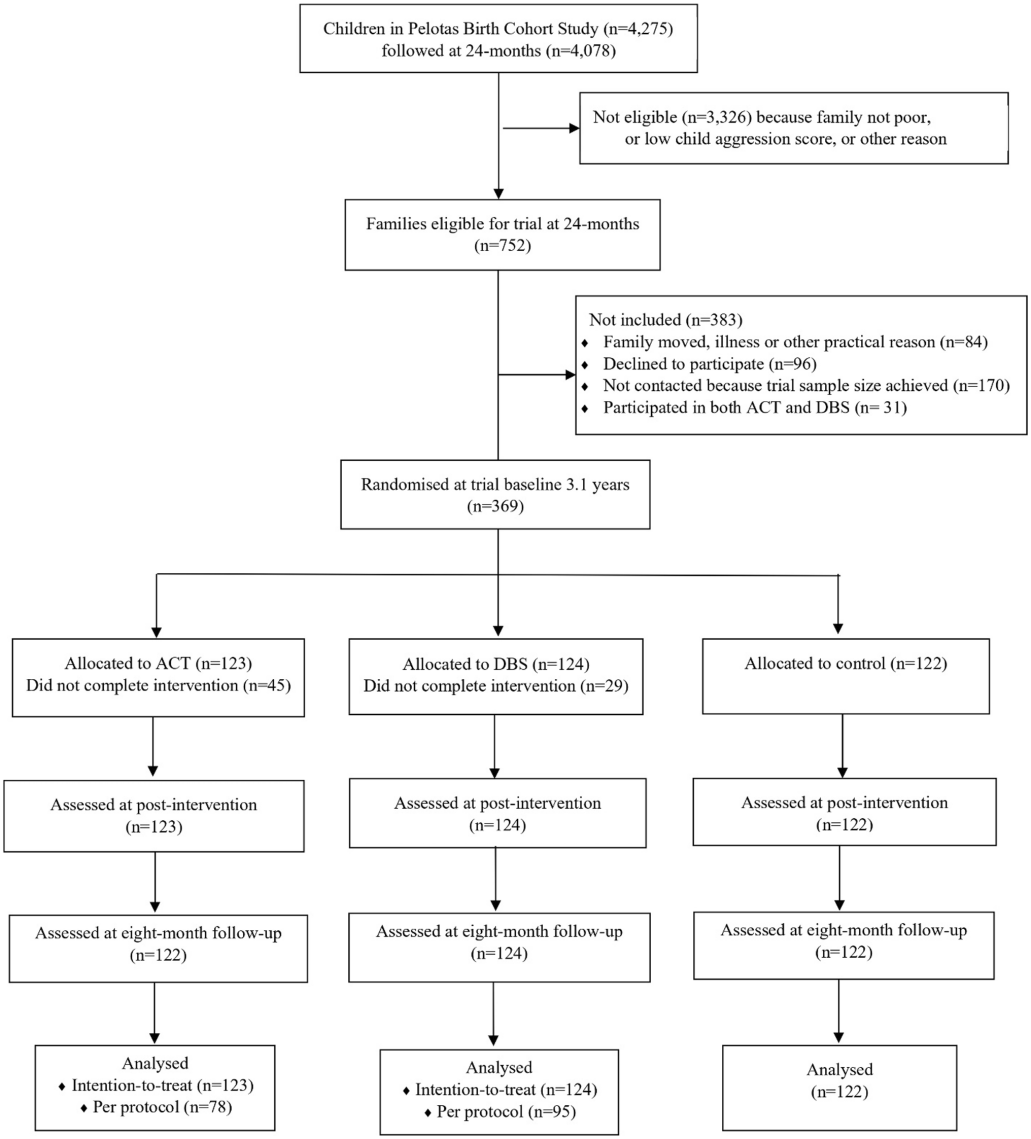
Consolidated standards of reporting trials diagram for the PIÁ Trial

Recruitment occurred between 18/6/2018 and 31/8/2018, and the last follow-up assessment was completed on 19/7/2019. Figure 1 shows participant flow in the study. The 369 participating mother–child pairs were assessed at baseline when children were mean 3.1 years (SD 0.31) and then randomised to ACT, DBS, or Control. At 1-month post-intervention, all 369 (100% of those included at baseline) were reassessed, and at 8-month follow-up 368 (99.7%) were again reassessed and included in ITT analyses.

Comparing the PIÁ trial sample to the general population (all children born in Pelotas in the same year) on age 24-month measures (before the trial) confirmed that the trial sample was high-risk in terms of poverty, low maternal education, maternal depression, coercive parenting, and child aggression (Supplementary Material, Table S8). Compared with the 752 families who had been identified as eligible for the trial, the actual PIÁ trial sample of 369 families was almost identical across all variables examined (Supplementary Material, Table S8).

Table 1 below shows key characteristics of the PIÁ sample at trial baseline (3.1 years), according to randomised group (ACT, DBS, or control), with about half of the children being boys, half being white, most not attending pre-school, and mothers having about 7 years of schooling, in each group. Tables S9 and S10 in the Supplement show additional participant characteristics and outcome scores at baseline by randomised group, with equivalence between them.

**Table 1:** Baseline characteristics of the intention-to-treat population in the PIÁ Trial

	**ACT (*n* = 123)**	**DBS (*n* = 124)**	**Control (*n* = 122)**
Child’s characteristics			
Child’s age (years)			
Mean (SD)	3.1 (0.3)	3.1 (0.3)	3.0 (0.3)
Child’s sex, *n* (%)			
Male	63 (51.2)	63 (50.8)	62 (50.8)
Female	60 (48.8)	61 (49.2)	60 (49.2)
Child’s skin colour, *n* (%)			
White	64 (52.0)	66 (53.2)	66 (54.1)
Black	24 (19.5)	28 (22.6)	26 (21.3)
Brown	30 (24.4)	28 (22.6)	29 (23.8)
Other	4 (3.3)	2 (1.6)	1 (0.8)
Missing	1 (0.8)	-	-
Attends school, *n* (%)			
No	80 (65.0)	88 (71.0)	89 (73.0)
Yes, full-time	35 (28.5)	30 (24.2)	22 (18.0)
Yes, part-time	7 (5.7)	6 (4.8)	11 (9.0)
Missing	1 (0.8)	-	-
Child books at home, *n* (%)			
Yes	87 (70.7)	95 (76.6)	94 (77.0)
No	35 (28.5)	27 (21.8)	28 (23.0)
Missing	1 (0.8)	2 (1.6)	-
Mother’s characteristics			
Mother’s age (years), *n* (%)			
Mean (SD)	27.1 (6.1)	28.9 (6.5)	28.8 (6.6)
Days spent with child (per week)			
Mean (SD)	6.2 (1.0)	6.2 (1.2)	6.1 (1.2)
Missing	2	-	-
Maternal education (years)			
Mean (SD)	7.5 (2.9)	7.3 (3.0)	7.5 (3.0)
Intimate partner violence, *n* (%)			
No	65 (52.8)	58 (46.8)	62 (50.8)
Yes	23 (18.7)	29 (23.4)	30 (24.6)
No partner	34 (27.6)	37 (29.8)	30 (24.6)
Missing	1 (0.8)	-	-
Maternal depression, *n* (%)			
Normal	78 (63.4)	65 (52.4)	75 (61.5)
Screened positive for depression	44 (35.8)	59 (47.6)	47 (38.5)
Missing	1 (0.8)	-	-

*ACT* Raising Safe Kids Program (ACT), *DBS* dialogic book-sharing, *SD* standard deviation, *JVQ* Juvenile victimization questionnaire

For ACT, 64% (*n* = 78) of participants completed the programme, and for DBS, 77% (*n* = 95); further details on adherence have been reported on previously (Martins et al., 2020). No cross-over was identified between arms.

**Fig. 2:**
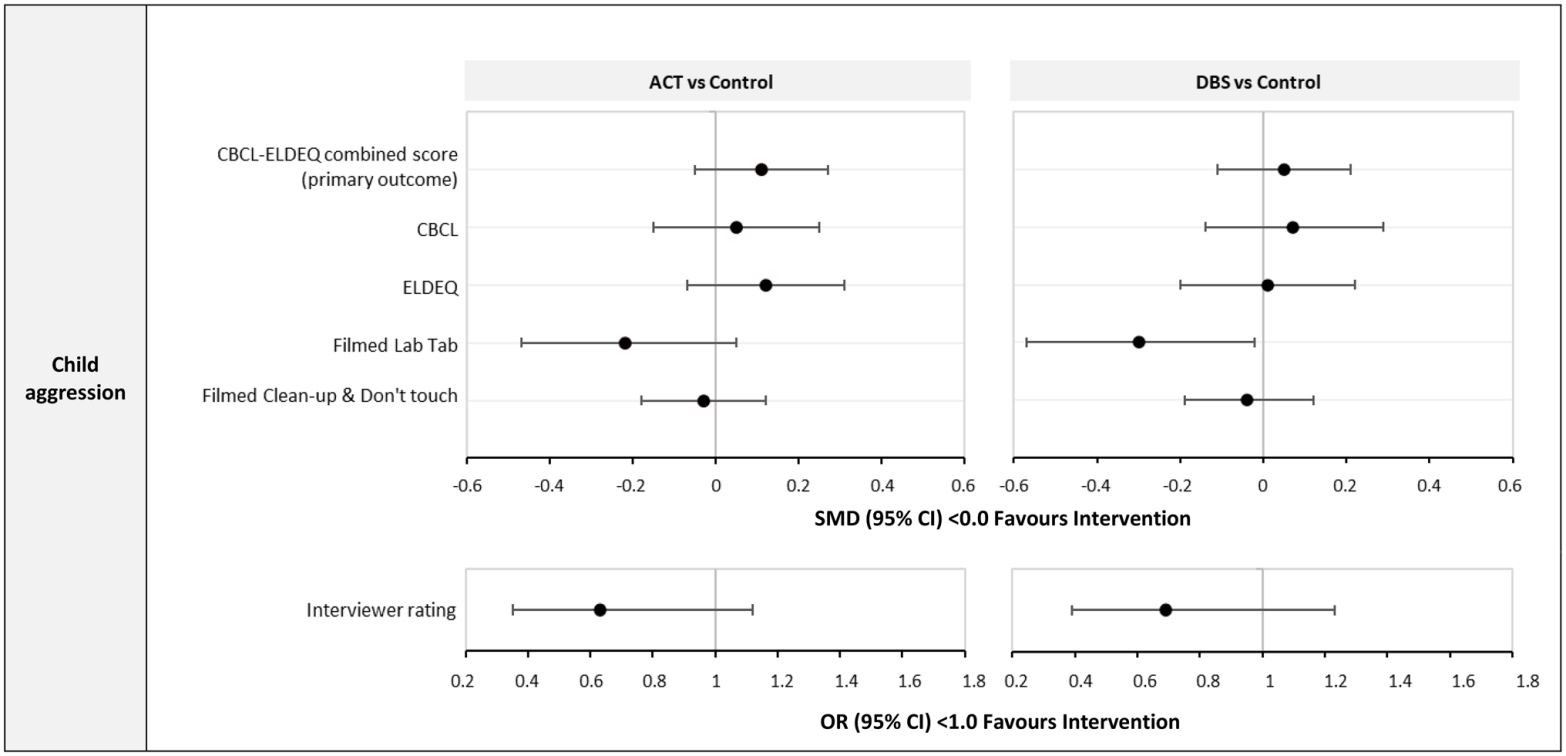
Effects of ACT and DBS on child aggression at 8 months follow-up. ACT, Raising Safe Kids Program; DBS, dialogic book-sharing programme; SMD, adjusted standardised mean difference between intervention group and control (in population standard deviation units); CI, confidence interval; OR, odds ratio

Figure 2 shows the adjusted SMDs for ACT and DBS for each child aggression outcome assessed using two questionnaires (CBCL and ELDEQ) and three observational measures (values are in the Supplement, Tables S8–S9). There was no evidence of effects of either intervention on the primary outcome (CBCL-ELDEQ combined) compared to the control: SMD 0.11 (95% CI − 0.05, 0.17) for ACT, and SMD 0.05 (95% CI − 0.11, 0.21) for DBS. There was no evidence that either intervention influenced other indicators of child aggression (all SMDs < 0.22, all 95% CIs including 0.0), except for a reduction in child anger on the Filmed LabTab task for DBS (SMD − 0.30; 95% CI − 0.58, − 0.03). Results from PP and sensitivity analyses including only generic covariates were similar to these ITT analyses (Supplement, Tables S12 and S17, respectively).

**Fig. 3:**
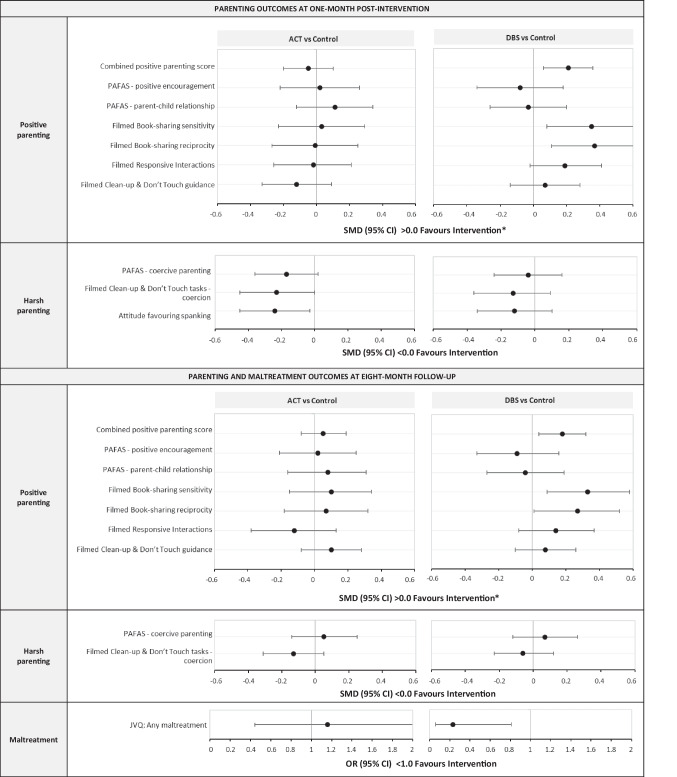
Effects of ACT and DBS on parenting and maltreatment outcomes at 1-month post-intervention, and 8-month follow-up. ACT, Raising Safe Kids Program; DBS, dialogic book-sharing programme; SMD, adjusted standardised mean difference between intervention group and control (in population standard deviation units); CI, confidence interval; OR, odds ratio; *For PAFAS positive encouragement and parent–child relationship, < 0.0 favours intervention

Figure 3 shows the effects of ACT and DBS on parenting and maltreatment outcomes (also Supplement Table S9). DBS was associated with an improvement in overall (combined) positive parenting, both 1 month (SMD 0.21; 95% CI 0.06, 0.36) and 8 months (SMD 0.18; 95% CI 0.04, 0.32) after the intervention. For specific aspects of positive parenting, the SMDs for DBS were 0.33 (95% CI 0.08, 0.57) for book-sharing sensitivity, and 0.27 (95% CI 0.02, 0.53) for book-sharing reciprocity at 8-month follow-up. The ACT intervention was associated with reduced favourable attitudes to spanking (SMD − 0.24; 95% CI − 0.45, − 0.03), and reduced maternal coercive behaviour in observations at 1-month post-intervention (filmed clean-up and do not touch tasks; SMD − 0.23; 95% CI − 0.46, − 0.01), but no difference was found at 8-month follow-up. DBS was associated with lower odds of maternal reports of maltreatment at the 8-month follow-up (OR 0.23; 95% CI 0.06, 0.81), but no other measure in the domain of harsh parenting. Neither intervention was associated with maternal reports of positive or harsh parenting on the PAFAS questionnaire (all SMDs < 0.20, all 95% CIs including 0.0). PP results for parenting outcomes were similar to ITT (Supplement Table 13). Exploratory, sensitivity analyses examined the effects of ACT on parenting/maltreatment, restricting the sample to participants who completed the course and did so with a high-fidelity facilitator (*n* = 45, Supplement Table S18). This showed one effect on positive parent–child relations reported on the PAFAS questionnaire at 8-month follow-up (SMD 0.35; 95% CI 0.04, 0.66), which should be treated cautiously given small numbers.

**Fig. 4:**
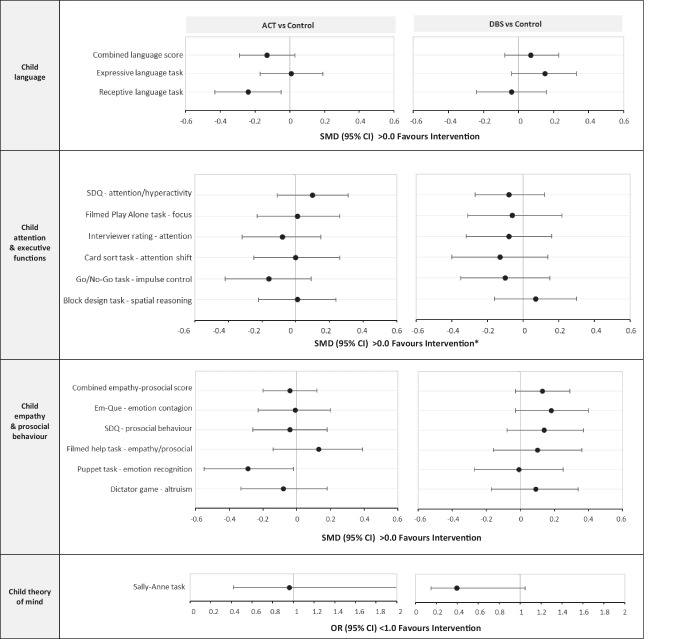
Effects of ACT and DBS on child development outcomes at 8-month follow-up. ACT, Raising Safe Kids Program; DBS, dialogic book-sharing programme; SMD, adjusted standardised mean difference between intervention group and control (in population standard deviation units); CI, confidence interval; OR, odds ratio. *For SDQ – attention/hyperactivity and interviewer rating, < 0.0 favours intervention

Figure 4 presents the effects of ACT and DBS on a range of child development outcomes (see also Supplement Table S14). There was no evidence of effects of either intervention on child language, apart from a negative effect of ACT on child receptive language (SMD − 0.24; 95% CI − 0.43, − 0.05). In PP analyses and sensitivity analyses, the results were similar (Supplement Tables S14 and S16). There was no evidence of effects of the interventions on child attention or executive function outcomes, empathy, prosocial behaviour, or theory of mind (all SMDs < 0.20, all 95% CIs including 0.0), except for one negative effect of ACT on the Puppet task measuring emotion recognition (SMD − 0.29; 95% CI − 0.56, − 0.03). In PP analyses, results were generally similar (Supplement, Table S14), apart from DBS improving child theory of mind (lower odds of failure on task: OR 0.22; 95% CI 0.06, 0.79), which should be cautiously interpreted given small numbers.

**Fig. 5:**
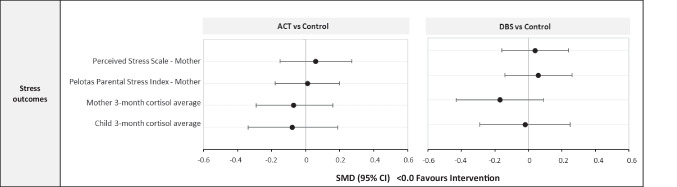
Effects of ACT and DBS on stress outcomes at 8-month follow-up. ACT, Raising Safe Kids Program; DBS, dialogic book-sharing programme; SMD, adjusted standardised mean difference between intervention group and control (in population standard deviation units); CI, confidence interval

The effects of the interventions on stress outcomes are shown in Fig. 5. No effect was identified for ACT or DBS on maternal reports of stress, or maternal or child hair cortisol concentrations (all SMDs < 0.2, all 95% CIs including 0.0), with PP analyses showing similar results (Supplement Table S15).

Moderator analyses investigated whether ACT or DBS might have differential effects according to child or maternal characteristics and baseline levels of outcomes. There was no evidence for differential effects (Supplement Table S19).

## Discussion

Supporting early child development and preventing violence are two mutually reinforcing major global agendas. Given the urgent need for more high-quality evidence about potentially scalable solutions in LMICs, we conducted a RCT of two low-cost parenting programmes, in a southern Brazilian city. Following key trial protocols, and using multiple methods of evaluation with a very high follow-up rate, some effects were identified on parenting, but no benefits were found on the primary outcome of child aggression, or other child outcomes relevant to the development of violence.

The only clear effects of ACT and DBS in the current trial were on caregiving practices directly taught in the interventions. DBS had a sustained effect on parental sensitivity and reciprocity during book-sharing, 8 months after the programme. A reduction in child maltreatment was also self-reported by mothers after DBS; however, other outcomes associated with maltreatment were not influenced by DBS, such as maternal harsh parenting or child behaviour, so without replication, this should be regarded as a chance finding. For ACT, there were reductions in observed harsh parenting and attitudes favourable to spanking soon after the intervention, but no benefits were found 8 months later in the main analyses. ACT’s aim to reduce violence against children, in terms of maltreatment, was not demonstrated.

The current trial did not find effects of either ACT or DBS on child aggression, the trial’s primary outcome. The most recent systematic review of parenting programmes in LMICs among children aged 2–17 years (Backhaus et al., 2023) found moderate-level evidence for reductions in conduct problems (as well as other benefits of such programmes, including reductions in harsh parenting); however, there was high heterogeneity across studies, and many had methodological weaknesses (Backhaus et al., 2023). Considering the lack of effects of ACT or DBS on child aggression in the current trial, it is relevant that, even for better-resourced and intensive interventions in HICs, it is not uncommon to find no or negligible effects of parenting programmes on child behaviour (Hendriks et al., 2018), and this is also true among several previous trials in LMICs (Backhaus et al., 2023; Burkey et al., 2018), including the only other trial that has examined child conduct problems as a pre-registered primary outcome (Lachman et al., 2017).

There are several possible reasons why ACT and DBS did not reduce child aggression or benefit other child outcomes in this trial. First, changes in carer-child interactions are the theorised mechanism by which such parenting programmes influence child development (Altafim et al., 2021; Murray et al., 2016a, b), and limited impact on parenting thus seems likely to explain lack of effects on child outcomes. That caregiving effects were observed only post-intervention for the ACT programme in the main analyses, and not at follow-up, is consistent with this explanation. In this trial sample, key parenting practices targeted by the programmes (coercive parenting and book-sharing practices), did correlate with child behaviour and language (Table S21); so the fact that the interventions did not achieve change in these child outcomes, is consistent with the finding that effects on parenting practices were limited. In the only other prior trial of ACT that evaluated child conduct problems (Altafim & Linhares, 2019), there were benefits (according to mothers, but not other caregivers) five weeks post-intervention, but there were no effects 8 months later in the current study, so fade out of effects is another possibility.

The null results for child aggression following DBS are consistent with one other trial of DBS in South Africa reporting no effects on child aggression (Dowdall et al., 2021), and also a trial of a separate early reading programme in Brazil (Weisleder et al., 2018). Unlike the current study though, prior evaluations of DBS found gains in child language and attention — important outcomes in their own right (Dowdall et al., 2021; Murray et al., 2023; Vally et al., 2015). The lack of benefits for these outcomes in the current study could explain why there were also no effects on child aggression, as DBS seems most likely to influence child aggression (if at all) via improvements in child language and cognition. As mentioned above, possibly DBS effects on maternal book-sharing practices were too small to influence these outcomes. In prior studies where DBS improved child cognition, larger effects on parental book-sharing practices were found (e.g., sensitivity SMD: 0.77 to 1.09; Dowdall et al., 2021; Murray et al., 2016a, b, 2023), compared to the current study (SMD: 0.35). Also, in the current study children were aged 3.1 years — older than in some prior studies of DBS, conducted when language development is more rapid (Dowdall et al., 2021; Murray et al., 2016a, b). Given that some effects on parental book-sharing practices were observed 8 months post-intervention in our study, changes in child outcomes might emerge later (sleeper effects), although some evidence in LMICs suggests effects on child cognition tend to fade out, rather than increase (Jeong et al., 2021).

Why were there not larger effects in this trial on parenting practices — the proximal targets of ACT and DBS? It is plausible that this relates to the particularly high-risk nature of the sample — poorer families with medium–high levels of child aggression at baseline, in a relatively poor city, with high levels of violence. For families in this context, dealing with other critical needs, such as food insecurity, a short parenting programme may not achieve greater impact without attending also to other basic needs. Although in HICs, intervention effects can be particularly clear for higher-risk samples (Olds et al., 2007), there is some evidence in LMICs that families in greatest risk may benefit less from parenting programmes (Aboud, 2007; Murray et al., 2016a, b). A second possible explanation for limited effects on parenting in this trial is that the interventions (not assessments) were implemented by non-researchers, which is likely to reduce the quality of implementation compared to a pure efficacy trial, despite strong researcher support given to maximise implementation quality in the current trial. A third possible reason for small effects is the level of parental adherence to the programmes (ACT 64%; DBS 77%), although this is not particularly low compared to other studies, and results in this trial were similar across per protocol and intention-to-treat analyses — so limited effects do not seem explained by levels of adherence. Finally, it is also possible that group-based programmes might achieve greater impact if additional direct support from a home visitor is also provided, which may be challenging in environments with limited resources.

Considering possible harms, in the current trial, two child measures (receptive language and emotion recognition) were worse in the ACT group compared with control. However, these were isolated results (other related measures did not have similar results), there was no hypothesised effect of ACT on these outcomes in either direction (Murray et al., 2019), and there is no theoretical reason why ACT caused such harms, particularly considering some positive effects, and no undesirable effects, on parenting outcomes.

These findings should be considered in relation to study limitations. Although the sample size was larger than originally planned, and larger than previous trials of ACT and DBS, power to detect small effects was still restricted, so some small benefits might not have been detected. Although a wide range of parenting and child outcomes were assessed with many self-report, observational, and biological measures, for the ACT programme, some other relevant outcomes, such as parental emotion regulation and child screen use, were not evaluated. Many outcomes were analysed to consider a wide range of possible intervention effects. This strengthens the conclusion that effects were limited (given such comprehensive outcome assessment); however, chance findings are more likely — for that reason, we focussed on the primary outcome and the few findings that were clinically plausible and consistent across measures, as recommended for such trials (Schulz & Grimes, 2005). The current trial sat between an efficacy and effectiveness study, evaluating ACT and DBS as implemented in routine government services, but with considerable researcher support to maximise implementation quality. Generalisability is therefore limited to this context, and ideally, effects would also be compared with those obtained under pure efficacy conditions, to identify whether null findings reflect core limitations of the programmes or implementation in a real-world setting. We report extensive data on parenting after the interventions, as is standard practice for such programmes that build up parenting skills in incremental steps (see also Table S20 in Supplement on book-sharing frequency). In future research, it could be of interest to also analyse the development of parenting skills during the programme, in which case implementation data might additionally be measured during the intervention. Ideally, the effects of a combined arm, including both ACT and DBS, would have been evaluated, but the implementation of both programmes together proved impractical, suggesting that this simultaneous combination is not relevant to practice anyway.

In conclusion, in an RCT with high quality, multi-method measures, a very high-follow-up rate, and careful implementation of interventions in a real-world, Brazilian setting, two parenting programmes had limited effects on caregiving practices, but no effects on child development outcomes. Considering programme theory of change, we conclude that both programmes can be implemented with good fidelity in routine services; parents engaged positively in the programmes, with reasonable adherence; some impact was found on parenting practices targeted by the interventions, but this was insufficient to translate into changes in child behaviour within an 8-month period. There is an overwhelming case to invest in programmes supporting nurturing care across the Global South, with millions of children failing to meet their developmental potential, and parenting programmes having important benefits, albeit with variable effects on child behaviour. Given ACT and DBS are short, low-cost programmes that are highly culturally transportable and have some support in other trials, they represent potential scalable solutions for LMICs. However, we find that, when delivered to a high-risk population, in routine services, even with strong research-team support, and endorsement by families themselves (Martins et al., 2020), benefits for child outcomes were not observed 8 months later. Because interventions with the most consolidated evidence base are often commercialised in HICs, with unrealistic costs for LMICs, there is an urgent need to continue evaluating programmes appropriate for LMICs, until replicated, positive effects are demonstrated and sustained in real-world settings.

### Supplementary Information

Below is the link to the electronic supplementary material.

Supplementary file1 (PDF 1263 KB)

## Data Availability

This trial is embedded in the 2015 Pelotas Birth Cohort study. Data access is given via submission of a paper proposal to a publications committee of the cohort, and contact about potential collaboration and access to data can be addressed to j.murray@doveresearch.org.

## Footnotes

1 Originally, there was a plan to evaluate the effects of ACT and DBS combined, but early implementation of this combination showed it was practically extremely difficult for participants, as well as facilitators, and thus the final trial design, as described in the protocol (Murray et al., 2019) before recruitment finished, included just three arms (ACT, DBS, and Control).
