# Supplementary material for: Effects of Two Early Parenting Programmes on Child Aggression and Risk for Violence in Brazil: a Randomised Controlled Trial
Source: Prev Sci. 2024 Jul 2;25(5):834–48. doi: 10.1007/s11121-024-01698-3 (PMC11322246; doi:10.1007/s11121-024-01698-3)
Supplement: Supplementary file 1 — Supplementary file1 (PDF 1263 KB) [file 11121_2024_1698_MOESM1_ESM.pdf]

Supplementary Material

Effects of two parenting programmes to reduce aggression and risk for violence in Brazil: a randomised controlled trial

Joseph Murray, Rafaela Costa Martins, Melanie Greenland, Suélen Cruz, Elisa Altafim, Adriane Xavier Arteche, Peter J. Cooper, Marlos Rodrigues Domingues, Andrea Gonzalez, Adriana Kramer Fiala Machado, Lynne Murray, Isabel Oliveira, Iná Santos, Tâmara Biolo Soares, Luciana Tovo-Rodrigues, Merryn Voysey
Prevention Science, 2024

Table of Contents

Description of the ACT Raising Safe Kids Program **3**

Table S1: ACT Raising Safe Kids Program intervention sessions content 5

Description of the DBS Book-sharing intervention **6**

Table S2 DBS Book-sharing intervention session content 8

Tables showing details of measures used in the PIÁ trial **9**

Table S3: Child aggression measures and timing of assessments 9

Table S4: Child development measures and timing of assessments 10

Table S5: Parenting and maltreatment measures, and timing of assessments 12

Table S6: Stress measures and timing of assessments 14

Table S7: Covariate measures and timing of assessments during birth cohort study or trial baseline 15

Tables showing characteristics of the PIÁ trial sample and other relevant populations **16**

Table S8: Characteristics (at age 24 months) of the 2015 Pelotas Birth Cohort, children eligible for the trial, and those included in the final PIÁ trial sample 16

Table S9: Additional baseline characteristics of the intention-to-treat population, by intervention group 18

Table S10: Baseline scores on trial outcomes of the intention-to-treat population, by intervention group 20

Tables showing results for the effects of ACT and DBS in the PIÁ trial **24**

Table S11: Unadjusted means for primary outcome of child aggression, and component scores, by intervention and time point 24

Table S12: Child aggression outcomes at 8-month follow-up and estimates of intervention effects 25

Table S13: Parenting and maltreatment outcomes and estimates of intervention effects 27

Table S14: Child development outcomes at 8-month follow-up and estimates of intervention effects 30

Table S15: Stress outcomes and estimates of intervention effects 33

Table S16: Child language outcomes at 8 month follow-up and estimates of intervention effects, using complete cases with valid baseline language data (exploratory, sensitivity analyses) 34

Table S17: Child aggression measures at 8-month follow-up, estimates of intervention effects adjusting for generic covariates only (exploratory, sensitivity analyses) 35

Table S18: Parenting outcomes for ACT estimates of intervention effects, restricting sample to participants who completed course with a good facilitator (exploratory, sensitivity analyses) 36

Table S19: Moderator analyses (intention-to-treat population) 38

Additional Information about Maternal Use of ACT and DBS Techniques Post-Intervention 42

Table S20: Descriptives of time implementing book-sharing in DBS group at one month post-intervention (N = 124) 42

Table S21: Correlations between key measures of ACT and DBS parenting techniques (post-intervention) and child outcomes (follow-up)....................43

Details of Processing of Hair for Cortisol Measurement 44

References for Supplement 45

Appendix A: CONSORT Checklist for the PIA trial 47

Appendix B: Pre-Specified Statistical Analysis Plan 49

Description of the ACT Raising Safe Kids Program

The ACT programme was developed by the American Psychological Association (APA) and is coordinated by the Children, Youth & Families Portfolio. The programme has been in operation since 2001, but underwent major redesign and review of its materials in 2006. ACT is a preventive intervention focusing on parents and caregivers of young children, and its purpose is to teach positive parenting skills and practices that help create stable, safe, healthy, nurturing environments and relationships that protect children from adverse experiences, such as abuse and neglect and their lifelong consequences. The core principles of the programme are that exposure to adverse childhood experiences (ACEs), such as maltreatment, especially in the early years, when children are learning foundational skills, can have serious and long-standing impacts on health, emotional, cognitive, and behavioural development. Parents and caregivers are teachers, protectors, and advocates for their children who can be supported to provide non-violent environments for their children to thrive and achieve their full potential.

The ACT programme consists of nine group-based sessions, preferably with a maximum of 10 parents/caregivers, delivered weekly in 2-hour sessions. Parents are given information (through interactive activities, as well as slides and videos) about strategies for: dealing with children's difficult behaviours using developmentally appropriate responses; controlling their own anger; helping children control their anger; teaching children how to resolve conflicts without using violence; using positive discipline methods that fit the child's age; and reducing the influence of media violence on children. The general themes of the ACT sessions are displayed in Table S1 below. For example, in the third session, guidance is given on how caregivers can understand and control their anger; and in the fourth session, guidance is provided on how to understand and help children when they feel angry.

ACT sessions have an interactive and experiential format, with activities that encourage interaction with the rest of the group, and engage participants in learning and practicing the content of the programme. In general, the sessions begin with a "warm-up" activity – related to the theme of the session and is usually made fun, aiming to engage parents in the session. The warm-up is followed by reviewing the homework from the previous session and explaining the purpose of the current session. After working on the central theme of the session, the content is summarised and parents receive homework to practice until the next session. The content of each intervention session is specified in the ACT Programme Facilitator User Manual, containing instructions on delivery and copies of the materials shown to parents. Parents received a handbook, containing fact sheets and handouts that help them learn about child development, how to deal with children's anger and challenging behaviours, and positive discipline strategies.

ACT facilitators are professionals with ideally a bachelor's degree in fields such as education, social work, psychology and early education, among others relevant to general knowledge of child development. For training and certification of ACT facilitators, a two-day in-person training workshop is given, conducted by a certified ACT coordinator/master trainer. The workshop prepares professionals to conduct the ACT programme with groups of parents and caregivers of young children. The ACT facilitator training workshop incorporates an interactive, experiential format to engage professionals in learning and practicing the content and concepts

of the program. The workshop includes a thorough review of the programme materials, and simulation of activities outlined in the curriculum, which participants would use with the groups of parents.

In the PIA trial, an experienced ACT master trainer (EA) conducted the two-day training workshop and liaised with the senior trial psychologist (SC) to provide support during the first practice group before ACT accreditation. Facilitators were primarily municipal school coordinators, and also some psychologists who were accredited in ACT before trial programme implementation. During the sessions, an ACT co-facilitator completed the session checklist ensuring that all the activities of the ACT programme had been conducted properly and, when necessary, reminding the facilitator of any activity needing completing or complementing the facilitator's statements according to the programme guide. Additionally, weekly supervision sessions were held with between the ACT facilitators and the senior psychologist member of the research team, who also participated in the ACT training workshop. Two of the ACT sessions were filmed for each facilitator, and a random one of the two evaluated by the senior psychologist in terms of fidelity to the programme (proportion of content correctly implemented during the session). The adherence of PIÁ participants to the ACT programme and their (positive) impressions about it have been reported on in a previous publication (Martins et al., 2020).

Table S1: ACT Raising Safe Kids Program intervention sessions content

Session	Session Content
1	**Pre-Meeting: Motivation and Behavioural Changes**<br>The benefits and objectives of the ACT program are explained and the rules of the group meetings are established. Parents are encouraged to think and discuss about the dreams they have for their children.
2	**Learning Child Development and Understanding Children's Behaviour**<br>Helping parents / caregivers learn basic elements of child development and how to respond appropriately to their children's behaviour.
3	**Young Children's Exposure to Violence**<br>Helping parents understand how children may be exposed to violence and the consequences it will have on their lives.
4	**Understanding and Controlling Parents/Adults' Anger**<br>Helping parents learn to control and deal with anger.
5	**Understanding and Helping Angry Children**<br>Helping parents understand children's feelings of anger, and learn how to teach them to control their feelings.
6	**Children and Electronic Media**<br>Helping parents understand the impact of electronic media on their children's behaviour, and show them some options on how to reduce children's exposure to violence.
7	**Discipline and Parenting Style**<br>Helping parents understand that the way they raise their children has an impact on their lifelong behaviour.
8	**Discipline for Positive Behaviours**<br>Teaching parents how to prevent difficult behaviours and how to use positive ways of disciplining children.
9	**Parents as Teachers, Protectors and Advocates at Home and in the Community**<br>Helping participants understand what they have learned from the ACT program, and that it is already helping them to make the dreams they have for their children come true. Encourage participants to use at home and in the community the tools they have learned and reinforce in parents their role as teachers, protectors and advocates for their children.

Description of the DBS Book-sharing intervention

The book-sharing intervention was an adaptation of one originally developed in the USA by Whitehurst et al. (1988) and shown to be of benefit in numerous studies, principally conducted in the US, to child language development (Dowdall et al., 2020). The version we used was previously delivered in South Africa, delivered to small groups of parents, weekly, over a period of seven weeks (Cooper et al., 2014; Murray et al., 2016; Vally, Murray, Tomlinson, & Cooper, 2015). This duration is at the upper end of the range of effective interventions in the Dowdall et al. (2020) review. The core principles are that parents are trained in how to support their child's interest and active engagement, rather than simply 'reading' to their child. Responsiveness is emphasized that is sensitive to the child's developmental capacity and experience, as well as the importance of a positive encouraging approach.

Twelve facilitators in Pelotas were trained to deliver the DBS programme. They were selected from a pool of facilitators working for the municipality delivering another home-based child development programme (PIM), and were undergraduate students. The DBS programme was initially brought to Pelotas by the programme developers (Lynne Murray and Peter J. Cooper) who worked with the local research team and municipal government to translate the materials for the local context, and train lead supervisors of the programme for local delivery. Facilitator training in DBS was delivered by David Jeffery of the Mikhulu Trust (www.mikhulutrust.org) in a 5-day course, with support from two supervisor researchers, who had also received prior DBS training. The training aimed to teach facilitators the content of the course, and how to deliver the course as well as possible, with high fidelity to the programme.

The weekly, facilitator-led small-group sessions were conducted with groups of under ten parents, during which children were cared for in an adjoining play space by Center staff. Group sessions lasted approximately 50 minutes and were followed by a brief period of one-to-one discussion between the facilitator and each parent (together with their child).

The intervention provided parents with guidance on how to share books with their child in ways that best support child development, based on research on the parenting predictors of child cognitive and socio-emotional functioning (e.g., use of cognitively enriching extensions to child utterances to promote language development (Taumoepeau, 2016), and of mental state talk to promote social understanding (see Devine & Hughes, 2018, for a meta-analytic review). Facilitators delivered the intervention to parent groups via Powerpoint presentations that included demonstration video examples of parents using good book-sharing practices with children. The content of each intervention session was specified in a facilitator user manual containing copies of the materials shown to parents, along with related instructions on delivery. Sessions first recapitulated the core learning points of previous ones, and then focused on a particular theme, using a designated children's book, ('the book of the week'), covering specific book-sharing techniques to enhance child development (see Table S2 for session books and content). For example, in the second session guidance was given on responding to children by building on what they say and making links between the book content and the child's own experience; and in the fourth session, guidance was provided on highlighting the book characters' emotional expressions and experiences.

During the group sessions, the facilitator invited parents to comment on the videos and to join in discussion about the recommended techniques. Sample illustrations from the week's book were included in the Powerpoint presentations and used to facilitate group discussion about how the techniques covered in the session could be applied when sharing the book with the child. The books used during training were selected to match each session theme and to provide opportunities to rehearse the sessions' techniques. For example, the fourth session concerning emotions used 'Hug', by Jez Alborourgh, a book about a baby monkey being separated and then reunited with its parent. At the end of each group session, in order to generalize skills across different books, the application of the DBS techniques covered that week were discussed in relation to the previous weeks' books. Books were either wordless, or text-light, since this format has been shown to elicit mutually responsive parent-child interactions, and to afford elaborated dialogic talk, including talk concerning the mental states of book-characters.

Following the group session, the facilitator met individually with each parent and their child for five to ten minutes, as required, and invited them to share the book of the week together, using the techniques covered in the group session. This provided an opportunity for the facilitator to encourage and support parents, as well as to model particular book-sharing techniques if parents were uncertain about them. Any challenges parents experienced in implementing book-sharing at home were discussed, and strategies for managing these explored. As noted, sessions recapitulated key points from the previous ones, thereby reducing the possible impact on participants of their having missed any given session. In addition, to further address this issue, if parents had missed a session, the facilitator made sure the relevant learning points were well-understood in the subsequent one-to-one meeting.

Each book of the week was given to participants to take home with them at the end of the session, along with a card containing brief reminders of the session's main points. Participants were encouraged to practice sharing the book with their child regularly over the coming week, and they reported back about their experiences at each group session. The adherence of PIÁ participants to DBS and their (positive) impressions about it have been reported on previously (Martins et al., 2020).

Table S2 DBS Book-sharing intervention session content

Session	Session Content
1	**Introduction to Book Sharing (using 'Handa's Surprise' by Eileen Browne)** The benefits to child development of book-sharing are explained, and the importance of establishing a book-sharing routine stressed. Basic principles of dialogic reading are outlined, including following the child's lead, as well as techniques such as pointing and naming, and asking 'who/what/where' questions to engage the child and encourage a dialogue
2	**Elaborating and Linking (using 'Little Helpers' by Lynne Murray, Peter Cooper, and Lyn Gilbert)** Picking up on the child's focus of interest and elaborating on it. Making links between the book content and the child's own experience. Making links between different elements of the book, and their relation to the overall book narrative.
3	**Numeracy and Comparisons (using 'Handa's Hen' by Eileen Browne)** Introducing the idea of counting and comparative concepts (e.g., more, less, highest, smallest), and category inclusion and exclusion.
4	**Talking about Feelings (using 'Hug' by Jez Alborough)** Talking about the feelings of the book characters. Naming feelings and contextualizing them. Linking the book characters' feelings to the child's own emotional experience.
5	**Talking about Intentions (using 'Harry the Dirty Dog' by Gene Zion and Margaret Bloy Graham)** Discussing why characters feel the way they do, asking what characters are thinking and intending, encouraging the child to be curious about what will come next in the story.
6	**Talking about Perspectives (using 'Harry by the Sea' by Gene Zion and Margaret Bloy Graham)** Helping the child understand that different people can see things differently, know different things, and feel differently about things.
7	**Relationships (using 'The Wrong Side of the Bed' by Edward Ardizzone)** Discussing family relationships, including conflict and resolution.
8	**Review session** Recapitulation of key principles of book sharing and discussion about how participants will take the book sharing ahead in their day-to-day lives.

Tables showing details of measures used in the PIÁ trial

Table S3: Child aggression measures and timing of assessments

Outcome	Measure	Details	Score range* Desirable score is higher (+) or lower (-)	Baseline	1 Month Post Intervention	8 Months Follow-Up
**Child aggression: combined measure (primary outcome)**	**CBCL-ELDEQ combined**<br>Combined score of:<br>• Child Behaviour Checklist – aggression subscale (Achenbach & Rescorla, 2000)<br>• ELDEQ study questionnaire - aggression (Côté, Boivin, Nagin, & et al., 2007)	See individual CBCL and ELDEQ measures below for details. The final combined score was calculated as the mean of the z-score on each questionnaire.	z-score (-)	✓		✓
**Child aggression: individual measures**	**CBCL**<br>Child Behaviour Checklist – aggression subscale (Achenbach & Rescorla, 2000)	Questionnaire completed by mother: 19 items of the aggression sub-scale are summed to form a final score.<br>$\alpha = 0.89$<br>$\omega = 0.90$	0-38 (-)	✓		✓
	**ELDEQ**<br>ELDEQ (Etude Longitudinale du Development des Enfants du Quebec) study questionnaire - aggression score (Côté et al., 2007)	Questionnaire completed by mother, with 9 items of the aggression sub-scale summed to form a final score. The questionnaire was forward translated from both English and French to Portuguese, finalised, and back translated to English for this study. Factor analyses of 14 items applied in the whole 2015 Pelotas Birth Cohort Study identified the most appropriate 9 items for the final score.<br>$\alpha = 0.86$<br>$\omega = 0.87$	0-17 (-)	✓		✓
	**Filmed LabTab**<br>Child response to the frustration task from the *Laboratory Temperament Assessment Battery* for preschool children (Gagne, 2019)	Child is left with a wrong key for a locked box with desirable toy inside. Levels of anger/frustration are coded for each 20 second interval, and the final score equals the mean across four minutes of observation.<br>ICC baseline = 0.99	0-4 (-)	✓		✓
	**Filmed Clean Up & Don't Touch**<br>Combined score of:<br>• Child defiant behaviour on filmed 'Don't touch' task (Kochanska & Aksan, 1995; Pereira, Negrão, Soares, & Mesman, 2014)<br>• Child defiant behaviour on filmed 'Clean up' task (NICHD, 1998; Pereira et al., 2014)	During the Don't Touch task toys are placed in front of the child and the child is prohibited from touching the toys for two and a half minutes. During the Clean Up Task lasting for about three minutes, children are asked to clean up toys, with maternal support. Child defiant behaviour is coded for each 20 second period during the tasks, and the maximum for each task is calculated before being converted to a z-score. The final combined score was calculated as the mean of the z-score on each task.<br>Weighted Kappa Don't Touch = 0.97<br>Weighted Kappa Clean Up baseline = 0.93	z-score (-)	✓		✓
	**Interviewer rating**<br>Interviewer score on child defiance item from the Dunedin Longitudinal Study (Caspi & Silva, 1995)	Based on the behaviour of the child throughout the entire assessment procedure, the interviewer gives a single rating of their perceptions of the child's level of defiance.<br>Weighted Kappa = 0.62	1-3 (-)	✓		✓

* The range of possible values on the instrument is shown (not observed range in dataset), with "+" indicating that higher scores are more desirable outcomes and "-" indicating that lower scores are more desirable (e.g., lower child aggression is regarded as a better outcome).

Table S4: Child development measures and timing of assessments

Outcome	Measure	Details	Score range* Desirable score is higher (+) or lower (-)	Baseline	1 Month Post Intervention	8 Months Follow-Up
**Child language**	**Combined language score**<br>Combined score of:<br>• Expressive language task (Capovilla, Negrão, & Damázio, 2011)<br>• Receptive language task (Capovilla et al., 2011)	See individual language measures below for details. The final combined score was calculated as the mean of the z-score on each task.	z-score (+)	✓		✓
	**Expressive language task**<br>Teste de Vocabulário Auditivo e Teste de Vocabulário Expressivo (Capovilla et al., 2011)	100 pictures are sequentially shown to the child who is asked to name them. Pictures are increasingly hard to name, and after 5 consecutive errors, the task was finalised. The final score is equal to total correctly named images.	0-100 (+)	✓		✓
	**Receptive language task**<br>Teste de Vocabulário Auditivo e Teste de Vocabulário Expressivo (Capovilla et al., 2011)	Five images are shown to the child and the child is asked to point to the image which the interviewer gives the name for. This is repeated 33 times and the final score is the sum of correct answers.	0-33 (+)	✓		✓
**Child attention and executive functions**	**SDQ - inattention/hyperactivity**<br>Attention/hyperactivity sub-scale on the Strengths and Difficulties Questionnaire (Goodman, 2001)	Questionnaire completed by mother, with 5 items on the inattention/hyperactivity sub-scale summed to form a final score.<br>$\alpha = 0.74$<br>$\omega = 0.75$	0-10 (-)	✓		✓
	**Filmed Play Alone task – focus**<br>Child focus during a filmed period of playing alone with toys (Cooper et al., 2014; Kannass, Oakes, & Shaddy, 2006)	The child is left to play with 3 toys on the table without mother or examiner interference. The extent of clear focus and purposeful play (e.g. putting one thing systematically in relation to another) is scored for each 30 second period for 2 and a half minutes, and a mean forms the score.<br>ICC baseline = 1.00	0-4 (+)	✓		✓
	**Interviewer rating - inattention**<br>Interviewer score on child inattention/lack of concentration item from the Dunedin Longitudinal Study (Caspi & Silva, 1995)	Based on the behaviour of the child throughout the entire assessment procedure, the interviewer gives a single rating of their perceptions of the child's level of inattention/lack of concentration.<br>Weighted Kappa = 0.60	1-3 (-)	✓		✓
	**Card Sort task – attention shift**<br>From the Early Years Toolbox (Howard & Melhuish, 2017)	Test administered on the iPad involving sorting rabbits and boats by colour and by shape, with shift capacity scores representing total correct sorting of objects.	0-12 (+)	✓		✓
	**Go/No-Go task – impulse control**<br>From the Early Years Toolbox (Howard & Melhuish, 2017)	Test administered on the iPad involving tapping the screen for "Go" stimuli (80%) and not tapping for "No-Go" stimuli (20%), with impulse control referring to product of proportional Go and No-Go accuracy.	0-1 (+)	✓		✓
	**Block Design task – spatial reasoning**<br>From the Wechsler Preschool and Primary Scale of Intelligence (Wechsler, Golombok, & Rust, 1992)	Child is given 10 tasks of recreating block designs presented to them, each scored 0-2, depending on whether blocks are assembled correctly on first or second attempts, and summed for the final score.	0-20 (+)			✓

Outcome	Measure	Details	Score range* Desirable score is higher (+) or lower (-)	Baseline	1 Month Post Intervention	8 Months Follow-Up
**Child empathy-prosocial behaviour**	**Combined empathy-prosocial score**<br>Combined score of:<br>• Em-Que questionnaire – Emotion Contagion (Rieffe, Ketelaar, & Wiefferink, 2010)<br>• Strengths and Difficulties Questionnaire - Prosocial behaviour score (Goodman, 2001)	See individual measures below for details. The final combined score was calculated as the mean of the z-score on each questionnaire.	z-score (+)	✓		✓
	**Em-Que – Emotion Contagion**<br>From the Em-Que empathy questionnaire - subscale on emotion contagion (Rieffe et al., 2010)	Questionnaire completed by the mother, with six items summed to form a final score.<br>$\alpha = 0.64$<br>$\omega = 0.65$	0-12 (+)	✓		✓
	**SDQ - Prosocial behaviour**<br>Strengths and Difficulties Questionnaire – Prosocial behaviour subscale (Goodman, 2001)	Questionnaire completed by the mother, with 5 items of the prosocial behaviour sub-scale summed to form a final score.<br>$\alpha = 0.55$<br>$\omega = 0.55$	0-10 (+)	✓		✓
	**Filmed Help task – empathy/prosocial score**<br>Filmed Help task – empathy/prosocial score (Buttelmann, Carpenter, & Tomasello, 2009)	The examiner feigns experiencing a problem (i.e., being unable to find something, in this case a glue stick that the examiner has enthusiastically been using, that is left in view of the child) and the child's response is observed. Child helping behaviour is scored with the highest score referring to picking up the lost object and returning it to the examiner.<br>Weighted Kappa = 0.98	0-3 (+)	✓		✓
	**Puppet task – emotion recognition**<br>Denham's Affect Knowledge task (Denham et al., 2014)	Photos of children's faces showing different emotional expressions (happy, sad, anger, and fear) are shown to the child on puppets, and the child is asked to identify the emotions by naming and pointing. Pictures previously used in Brazil from the Child Affective Facial Expression (CAFE; LoBue & Thrasher, 2015) were selected.	0-4 (+)	✓		✓
	**Dictator Game – altruism**<br>Dictator game task – altruism score (Benenson, Pascoe, & Radmore, 2007)	The child is shown 10 stickers that they are told they can keep, before learning the stickers are finished, so not everyone will be able to receive 10 stickers. The child is left to divide the stickers between boxes for him/herself and the next child, and the number of stickers provided for the next child are counted.	0-10 (+)			✓
**Theory of Mind**	**Triangle task** (Abell, Happé, & Frith, 2000)	Computer-presented animations of geometric shapes (one large red and one small blue triangle) are presented and the child is questioned about what happened. Responses are transcribed and coded as perceptions of random, goal-directed, or mental state-related activity.	0-2 (+)	✓		
	**Sally-Anne task** (Baron-Cohen, Leslie, & Frith, 1985)	Two puppets are shown to the child with a ball and basket, with one puppet not seeing a change in position of the ball. Children are asked where the puppet who did not observe the change will think the ball is.	0-1 (-)			✓

* The range of possible values on the instrument is shown (not observed range in dataset), with "+" indicating that higher scores are more desirable outcomes and "-" indicating that lower scores are more desirable (e.g., higher empathy is regarded as a better outcome).

Table S5: Parenting and maltreatment measures, and timing of assessments

Outcome	Measure	Details	Score range* Desirable score = higher (+) or lower (-)	Baseline	1 Month Post Intervention	8 Months Follow-Up
**Positive parenting**	**Combined positive parenting score**<br>Combined score of:<br>• Filmed Responsive Interactions – sensitivity<br>• Filmed book-sharing - sensitivity<br>• Filmed book-sharing task – reciprocity<br>• Filmed Don't touch – Guidance<br>• Filmed Clean Up – Guidance	See individual measures below for details. The final combined score was calculated as the mean of the z-score on each task.	z-score (+)	✓	✓	✓
	**PAFAS - positive encouragement**<br>PAFAS questionnaire positive encouragement subscale (Sanders, Morawska, Haslam, Filus, & Fletcher, 2014)	Questionnaire completed by the mother. A factor analysis of the questionnaire applied in the whole cohort showed that the original three-item positive encouragement sub-scale should be reduced to two items, which were summed to form a final score.<br>α = 0.65<br>ω = 0.75	0-6 (-)	✓	✓	✓
	**PAFAS – parent-child relationship subscale**<br>PAFAS questionnaire parent-child subscale (Sanders et al., 2014)	Questionnaire completed by the mother. Five items of the relationship sub-scale are summed to form a final score.<br>α = 0.84<br>ω = 0.84	0-15 (-)	✓	✓	✓
	**Filmed Book-sharing task – sensitivity**<br>Filmed Book-sharing task – parental sensitivity score (Cooper et al., 2014; Murray et al., 2016; Vally et al., 2015)	Mother and child are filmed for approximately 5 minutes without examiner interference while looking at a picture book together. Subsequently, reviewers study the filmed interaction and code mother's and children's behaviours.<br>Weighted Kappa = 0.98	1-5 (+)	✓	✓	✓
	**Filmed Book-sharing task – reciprocity**<br>Filmed Book-sharing task – parent-child reciprocity score (Cooper et al., 2014; Murray et al., 2016; Vally et al., 2015)	Mother and child are filmed for approximately 5 minutes without examiner interference while looking at a picture book together. Subsequently, reviewers study the filmed interaction and code mother's and children's behaviours.<br>Weighted Kappa = 1.00	1-5 (+)	✓	✓	✓
	**Filmed Responsive Interactions**<br>Responsive Interactions task – overall responsive interactions score (Prime et al., 2015; Schneider et al., 2021)	The mother is instructed to sit and play with her child for five minutes constructing a robot as per pictures shown, using play (Lego/Duplo type blocks). Raters code 11 items about communicative clarity, mutuality building and mind-reading, on a 5-point Likert scale, and the mean represents the total responsive interactions score.<br>α cohort = 0.92<br>ω cohort = 0.94<br>ICC cohort= 0.62	1-5 (+)	✓	✓	✓

Outcome	Measure	Details	Score range* Desirable score = higher (+) or lower (-)	Baseline	1 Month Post Intervention	8 Months Follow-Up
	**Filmed Clean Up & Don't Touch tasks - guidance**<br>Combined score of:<br>• Parental guidance on filmed 'Don't touch' task (Kochanska & Aksan, 1995; Pereira et al., 2014)<br>• Parental guidance on filmed 'Clean up' task (NICHD, 1998; Pereira et al., 2014)	During the Don't Touch task toys are placed in front of the child and the child is prohibited from touching the toys for three minutes. During the Clean Up Task lasting for about three minutes, children are asked to clean up toys, with maternal support. Maternal supportive physical and verbal guidance is coded for each 20 second period during the tasks, and the average represents is calculated on each task before being transformed to a z-score. The final combined score was calculated as the mean of the z-score on each task.<br>ICC Don't Touch = 0.99<br>Weighted Kappa Clean Up baseline = 1.00	z-score (+)	✓	✓	✓
**Harsh parenting**	**PAFAS - coercion**<br>PAFAS questionnaire parental coercion subscale (Sanders et al., 2014)	Questionnaire completed by the mother. A factor analysis of the questionnaire applied in the whole cohort showed that the original five-item coercion sub-scale should be reduced to four items, which were summed to form a final score.<br>α = 0.70<br>ω = 0.71	0-12 (-)	✓	✓	✓
	**Filmed Clean Up & Don't Touch tasks - coercion**<br>Combined score of:<br>• Parental coercion on filmed 'Don't touch' task (Kochanska & Aksan, 1995; Pereira et al., 2014)<br>• Parental coercion on filmed 'Clean up' task (NICHD, 1998; Pereira et al., 2014)	During the Don't Touch task toys are placed in front of the child and the child is prohibited from touching the toys for three minutes. During the Clean Up Task lasting for about three minutes, children are asked to clean up toys, with maternal support. Maternal coercive behaviour is coded for each 20 second period during the tasks, and the maximum is calculated and then was transformed to a z-score. The final combined score was calculated as the mean of the z-score on each task.<br>Weighted Kappa Don't Touch = 0.99<br>Weighted Kappa Clean Up baseline = 0.94	z-score (-)	✓	✓	✓
**Attitudes about punishment**	**Attitude favouring spanking**<br>Child Development Project questionnaire on attitudes to physical punishment (Deater-Deckard, Lansford, Dodge, Pettit, & Bates, 2003)	Questionnaire completed by mother, with 5 items of the 5 sub-scale summed to form a final score.<br>α baseline= 0.78<br>ω baseline = 0.79	1-5 (-)	✓	✓	
**Maltreatment**	**JVQ – maltreatment**<br>Juvenile Victimization Questionnaire: 2nd Revision (JVQ-R2), maltreatment composite score (Finkelhor, Hamby, Ormrod, & Turner, 2005; Finkelhor, Hamby, Turner, & Ormrod, 2011)	Questionnaire completed by mother, with five items from the maltreatment sub-scale summed and dichotomised to form a final score.	0-1 (-)	✓		✓

* The range of possible values on the instrument is shown (not observed range in dataset), with "+" indicating that higher scores are more desirable outcomes and "-" indicating that lower scores are more desirable (e.g., lower harsh parenting is regarded as a better outcome).

Table S6: Stress measures and timing of assessments

Outcome	Measure	Details	Score range* Desirable score = higher (+) or lower (-)	Baseline	1 Month Post Intervention	8 Months Follow-Up
**Maternal stress**	**Perceived Stress Scale – Mother** Perceived Stress Scale completed by mother (Luft, Sanches, Mazo, & Andrade, 2007)	Questionnaire completed by mother, with 10 items summed to form a final score. α = 0.87 ω = 0.90	0-40 (-)	✓	✓	
	**Pelotas Parenting Stress Index – Mother** Research team adaptation of Parenting Stress Index (Abidin, 1997) completed by mother	Questionnaire completed by mother, with 8 items summed to form a final score. α = 0.78 ω = 0.77	0-24 (-)	✓	✓	
**Maternal cortisol**	**Mother 3-month cortisol concentration average** 3-month cortisol levels from mother's hair (pg/mg)	Hair samples provide a measure of cortisol concentration average over a three-month period and represent a measure of chronic levels of cortisol (and thus "toxic stress"), unlike other types of samples, such as saliva, blood, or urine which are used to measure acute cortisol concentrations at the time of collection. Hair cortisol was measured using a standardized protocol of hair cutting and storage, washing, grinding, hormone extraction and quantification (see Supplement)	pg/mg (-)	✓		✓
**Child cortisol**	**Child 3-month cortisol concentration average** 3-month cortisol levels from children's hair (pg/mg)	See maternal cortisol measure for details.	pg/mg (-)	✓		✓

* The range of possible values on the instrument is shown (not observed range in dataset), with "+" indicating that higher scores are more desirable outcomes and "-" indicating that lower scores are more desirable (e.g., lower stress is regarded as a better outcome).

Table S7: Covariate measures and timing of assessments during birth cohort study or trial baseline

Covariate	Measure	Score range*	Cohort perinatal	Cohort 24-month	Trial baseline
**Neighbourhood**	**Trial neighbourhood defined by study team**<br>11 geographic regions in Pelotas city were organised for the trial according to facilities to implement interventions and transport access for families	1-11			✓
**Maternal education**	**Maternal self-report in sociodemographic questionnaire**<br>Number of years of completed schooling in four categories	0-4	✓		
**Maternal skin colour**	**Maternal self-report in sociodemographic questionnaire**<br>Categories of skin colour (white, black, brown, yellow, indigenous, or other)	0-5			✓
**Family income**	**Maternal self-report in sociodemographic questionnaire**<br>Monthly family income in Brazilian reais (BRL)	0-1500		✓	
**Mother relationship status**	**Maternal self-report in sociodemographic questionnaire**<br>Categories of relationship status (with or without partner)	0-1			✓
**Intimate partner violence**	**Multi-country study questionnaire on women's health and violence against women (VAW) of the WHO** (Garcia-Moreno, Jansen, Ellsberg, Heise, & Watts, 2006)	0-13			✓
**Maternal depression**	**Edinburgh Postnatal Depression Scale (EPDS)** (Santos et al., 2007)	0-30			✓
**Maternal problem drinking**	**Alcohol Use Disorders Identification Test (AUDIT)** (World Health Organization, 2001)	0-1			✓
**Time mother spends with child per week**	**Maternal self-report in sociodemographic questionnaire**<br>In hours	0-168			✓
**Frequency reading/storytelling**	**Maternal self-report in sociodemographic questionnaire**<br>Frequency of storytelling past week (never, once or twice, three or four times, five times or more)	0-3 (+)		✓	
**Child at preschool**	**Maternal self-report in sociodemographic questionnaire**<br>Yes or no	0-1			✓
**Child callous/unemotional traits**	**Inventory of Callous and Unemotional Traits** (Ezpeleta, de la Osa, Granero, Penelo, & Domènech, 2013)	0-72			✓

* The range of possible values on the instrument is shown (not observed range in dataset), with "+" indicating that higher scores are better and "-" indicating that lower scores are better (e.g., lower maternal depression is regarded as better than high maternal depression). N/A = Not Applicable. Note, not all covariates were adjusted for, for every outcome. Covariates included in each model are listed in footnotes in Tables S8-S14, and summarised in Appendix Table 2.

Tables showing characteristics of the PIÁ trial sample and other relevant populations

Table S8: Characteristics (at age 24 months) of the 2015 Pelotas Birth Cohort, children eligible for the trial, and those included in the final PIÁ trial sample

	Total cohort (4,275)	Eligible for trial (N=752)	Trial sample (N=369)
Child’s characteristics			
Child's sex, n (%)			
Male	2164 (50.6)	387 (51.5)	188 (51.0)
Female	2111 (49.4)	365 (48.5)	181 (49.0)
Missing	0	0	0
Child's age (years)			
Mean (SD)	2.0 (0.1)	2.0 (0.1)	2.0 (0.5)
Missing	425	0	0
Child's skin colour, n (%)			
White	2829 (72.4)	423 (58.3)	215 (58.6)
Black	386 (9.9)	112 (15.4)	60 (16.4)
Brown	685 (17.5)	191 (26.3)	92 (25.1)
Other	6 (0.2)	0 (0.0)	0 (0.0)
Missing	369	26	2
Attends preschool, n (%)			
Yes	1071 (26.7)	111 (14.8)	56 (15.2)
No	2940 (73.3)	641 (85.2)	313 (84.8)
Missing	264	0	0
Child aggression score (0-9) on 3-item ELDEQ short scale			
Mean (SD)	2.3 (1.6)	3.3 (1.3)	3.3 (1.4)
Missing	266	0	0
Child language score			
Mean (SD)	33.9 (7.9)	33.8 (6.8)	33.8 (6.7)
Missing	421	16	7
Family income and mother’s characteristics			
Family income (R$ per month)			
Mean (SD)	3508.2 (5427.2)	944.1 (415.2)	929.6 (411.6)
Missing	327	0	0
Mother's age (years), n (%)			
Mean (SD)	27.7 (6.6)	25.2 (6.6)	25.2 (6.5)
Missing	1	0	0
Maternal education (years)			
Mean (SD)	10.1 (4.0)	7.4 (3.1)	7.4 (3.0)
Missing	1	0	0
Mother with a partner, n (%)			

	Total cohort (4,275)	Eligible for trial (N=752)	Trial sample (N=369)
Yes	3212 (84.6)	551 (73.3)	276 (74.8)
No	584 (15.4)	201 (26.7)	93 (25.2)
Missing	476	0	0
Maternal depression, n (%)			
Screened positive for depression	1074 (28.4)	344 (45.3)	169 (45.8)
Normal	2714 (71.7)	408 (54.3)	200 (54.2)
Missing	487		0
PAFAS questionnaire - coercive subscale			
Mean (SD)	4.2 (2.5)	5.0 (2.8)	5.0 (2.9)
Missing	489	0	0

SD, standard deviation

Table S9: Additional baseline characteristics of the intention-to-treat population, by intervention group

	ACT (N=123)	DBS (N=124)	Control (N=122)
Child’s characteristics			
Child's age (years), n (%)			
<=3	54 (43.9)	54 (43.5)	56 (45.9)
>3	69 (56.1)	70 (56.5)	66 (54.1)
Number of older aged siblings, n (%)			
0	56 (45.5)	48 (38.7)	51 (41.8)
1	49 (39.8)	42 (33.9)	40 (32.8)
2 or more	17 (13.8)	34 (27.4)	31 (25.4)
Missing	1 (0.8)	0 (0.0)	0 (0.0)
Child's use of corticosteroids, n (%)			
Yes	20 (16.3)	20 (16.1)	18 (14.8)
No	101 (82.1)	102 (82.3)	100 (82.0)
Missing	2 (1.6)	2 (1.6)	4 (3.3)
Reading-storytelling at 24 months, n (%)			
No	63 (51.2)	71 (57.3)	71 (58.2)
Yes, once or twice	31 (25.2)	27 (21.8)	27 (22.1)
Yes, three or more times	28 (22.8)	25 (20.2)	23 (18.9)
Missing	1 (0.8)	1 (0.8)	1 (0.8)
Number of books			
Mean (SD)	8.1 (13.3)	7.4 (9.0)	7.5 (7.3)
Missing	36	29	28
Carer during daytime (if not attending school), n (%)			
Mother	73 (91.3)	82 (93.2)	77 (86.5)
Other relative/sibling	7 (8.8)	6 (6.8)	12 (13.5)
Inventory of Callous-Unemotional Traits final score			
Mean (SD)	21.5 (9.6)	20.6 (8.8)	20.7 (9.1)
Range	(1.0, 53.0)	(4.0, 41.0)	(0.0, 44.0)
Missing	1	0	0
Mother’s characteristics			
Mother's age (years), n (%)			
<25	59 (48.0)	36 (29.0)	39 (32.0)
≥25 to <35	51 (41.5)	59 (47.6)	62 (50.8)
≥35	13 (10.6)	29 (23.4)	21 (17.2)
Mother's skin colour, n (%)			
White	59 (48.0)	72 (58.1)	62 (50.8)
Black	39 (31.7)	29 (23.4)	37 (30.3)
Brown	23 (18.7)	20 (16.1)	22 (18.0)
Other	1 (0.8)	3 (2.4)	1 (0.8)
Missing	1 (0.8)	0 (0.0)	0 (0.0)
Mother's use of corticosteroids, n (%)			
No	118 (95.9)	119 (96.0)	114 (93.4)

	ACT (N=123)	DBS (N=124)	Control (N=122)
Yes	3 (2.4)	4 (3.2)	6 (4.9)
Missing	2 (1.6)	1 (0.8)	2 (1.6)
Hours spent with child on a week day (per day)			
Mean (SD)	20.5 (4.6)	20.3 (5.2)	20.2 (5.3)
Range	(5, 24)	(2, 24)	(6, 24)
Missing	2	0	0
Hours spent with child on a weekend (per day)			
Mean (SD)	23.4 (3.2)	23.3 (3.0)	22.6 (3.8)
Range	(0, 24)	(0, 24)	(3, 24)
Missing	1	0	0
Maternal education (years), n (%)			
0-4	18 (14.6)	22 (17.7)	24 (19.7)
5-8	58 (47.2)	61 (49.2)	51 (41.8)
9-11	39 (31.7)	32 (25.8)	42 (34.4)
12 or more	8 (6.5)	9 (7.3)	5 (4.1)
Mother with a partner, n (%)			
Yes	88 (72.1)	87 (70.2)	92 (75.4)
No	34 (27.6)	37 (29.8)	30 (24.6)
Missing	1 (0.8)	0 (0.0)	0 (0.0)
Partner's relationship to child, n (%)			
Biological father of the child	79 (89.8)	76 (87.4)	80 (87.0)
Not the biological father of the child	9 (10.2)	11 (12.6)	12 (13.0)
Maternal drinking problem, n (%)			
Not harmful alcohol use	98 (79.7)	101 (81.5)	118 (96.7)
Harmful alcohol use	1 (0.8)	6 (4.8)	2 (1.6)
Missing	24 (19.5)	17 (13.7)	2 (1.6)
Neighbourhood in Pelotas (for interventions), n (%)			
Areal	10 (8.1)	11 (8.9)	17 (13.9)
Centro 1	12 (9.8)	5 (4.0)	16 (13.1)
Centro 2	8 (6.5)	14 (11.3)	8 (6.6)
Dunas	19 (15.4)	16 (12.9)	11 (9.0)
Fragata 1	11 (8.9)	12 (9.7)	11 (9.0)
Fragata 2	11 (8.9)	10 (8.1)	9 (7.4)
Getúlio/Pestano	11 (8.9)	11 (8.9)	11 (9.0)
São Gonçalo	8 (6.5)	12 (9.7)	6 (4.9)
Santa Terezinha	10 (8.1)	12 (9.7)	12 (9.8)
Simões Lopes/Padre Réus	10 (8.1)	11 (8.9)	12 (9.8)
Três Vendas	13 (10.6)	10 (8.1)	9 (7.4)

ACT: Raising Safe Kids Program (ACT); DBS: dialogic book-sharing programme; SD: standard deviation; JVQ: Juvenile Victimization Questionnaire

Table S10: Baseline scores on trial outcomes of the intention-to-treat population, by intervention group

	ACT (N=123)	DBS (N=124)	Control (N=122)
Child aggression - combined measure (z-score)			
Mean (SD)	-0.0 (0.9)	-0.1 (0.9)	0.1 (0.9)
Range	(-2.1, 2.5)	(-1.9, 2.2)	(-1.9, 2.4)
Missing	1	0	0
Child aggression - individual measures			
Child Behaviour Checklist - aggression subscale			
Mean (SD)	17.5 (8.3)	17.6 (8.3)	18.7 (7.7)
Range	(1.0, 38.0)	(0.0, 34.0)	(0.0, 35.0)
Missing	1	0	0
ELDEQ study questionnaire aggression score			
Mean (SD)	5.8 (4.0)	5.4 (3.6)	6.0 (3.6)
Range	(0.0, 17.0)	(0.0, 17.0)	(0.0, 17.0)
Missing	4	0	0
Filmed LabTab - aggression score			
Mean (SD)	0.5 (0.3)	0.5 (0.3)	0.5 (0.4)
Range	(0.0, 1.8)	(0.0, 2.2)	(0.0, 1.7)
Missing	3	1	2
Combined score (mean of z-scores) of:<br>Filmed 'Don't touch' - child behaviour<br>Filmed 'Clean Up' - child behaviour			
Mean (SD)	-0.0 (0.7)	0.0 (0.8)	0.0 (0.8)
Range	(-1.0, 2.0)	(-1.0, 2.0)	(-1.0, 2.5)
Missing	0	1	0
Interviewer rating - child defiance			
Nothing	85 (69.1)	76 (61.3)	83 (68.0)
A bit	31 (25.2)	36 (29.0)	23 (18.9)
A lot	7 (5.7)	11 (8.9)	15 (12.3)
Missing	0	1 (0.8)	1 (0.8)
Child language			
Combined score (mean of z-scores)			
Mean (SD)	-0.0 (0.9)	-0.1 (1.0)	0.0 (0.9)
Range	(-2.1, 1.9)	(-2.1, 1.9)	(-1.6, 2.1)
Missing	37	22	33
Expressive language task			
Mean (SD)	30.1 (18.4)	30.7 (19.7)	32.8 (18.8)
Range	(1.0, 67.0)	(0.0, 72.0)	(0.0, 79.0)
Missing	39	24	39
Receptive language task			
Mean (SD)	18.7 (6.4)	17.8 (6.3)	18.6 (5.3)
Range	(3.0, 31.0)	(3.0, 30.0)	(6.0, 31.0)
Missing	40	24	34

	ACT (N=123)	DBS (N=124)	Control (N=122)
Child attention and executive functions			
Strengths and Difficulties Questionnaire - attention/hyperactivity subscale			
Mean (SD)	5.5 (2.7)	5.6 (2.8)	5.7 (2.7)
Range	(0.0, 10.0)	(0.0, 10.0)	(0.0, 10.0)
Missing	1	0	0
Filmed Play Alone task - focus score			
Mean (SD)	2.2 (0.8)	2.3 (0.7)	2.3 (0.7)
Range	(0.0, 4.0)	(0.0, 4.0)	(0.0, 3.6)
Missing	0	1	1
Interviewer rating - child attention			
Mean (SD)	1.4 (0.6)	1.5 (0.6)	1.5 (0.6)
Range	(1.0, 3.0)	(1.0, 3.0)	(1.0, 3.0)
Missing	0	1	1
Go/No-Go task			
Mean (SD)	0.3 (0.2)	0.3 (0.2)	0.2 (0.1)
Range	(0.0, 1.0)	(0.0, 1.0)	(0.0, 0.9)
Missing	7	9	8
Block Design task			
Mean (SD)	1.6 (1.9)	2.0 (2.4)	1.4 (1.8)
Range	(0.0, 9.0)	(0.0, 9.0)	(0.0, 8.0)
Missing	0	4	3
Child empathy-prosocial behaviour			
Combined score (mean of z-scores)			
Mean (SD)	0.1 (0.8)	-0.0 (0.7)	-0.1 (0.8)
Range	(-1.9, 2.1)	(-3.1, 1.7)	(-2.2, 1.8)
Missing	1	0	0
Em-Que questionnaire - Emotion Contagion			
Mean (SD)	3.9 (2.6)	3.6 (2.4)	3.7 (2.6)
Range	(0.0, 12.0)	(0.0, 11.0)	(0.0, 12.0)
Missing	1	0	0
Strengths and Difficulties Questionnaire - Prosocial behaviour			
Mean (SD)	8.6 (1.5)	8.5 (1.5)	8.3 (1.7)
Range	(4.0, 10.0)	(1.0, 10.0)	(4.0, 10.0)
Missing	1	0	0
Filmed Help Task			
Mean (SD)	1.1 (1.2)	1.0 (1.1)	1.1 (1.1)
Range	(0.0, 3.0)	(0.0, 3.0)	(0.0, 3.0)
Missing	3	5	7
Denham's puppet task			
Mean (SD)	1.2 (0.8)	1.3 (0.7)	1.2 (0.8)
Range	(0.0, 3.5)	(0.0, 3.5)	(0.0, 3.5)

	ACT (N=123)	DBS (N=124)	Control (N=122)
Missing	13	13	12
Theory of Mind			
Triangle task			
Mean (SD)	0.3 (0.5)	0.2 (0.5)	0.2 (0.4)
Range	(0.0, 2.0)	(0.0, 2.0)	(0.0, 1.0)
Missing	30	31	48
Positive parenting			
Combined score (mean of z-scores)			
Mean (SD)	0.4 (0.6)	0.4 (0.6)	0.4 (0.6)
Range	(-0.7, 1.7)	(-1.0, 1.9)	(-1.5, 1.9)
Missing	0	1	0
PAFAS - positive encouragement subscale			
Mean (SD)	0.8 (1.0)	0.9 (1.0)	0.7 (0.9)
Range	(0.0, 6.0)	(0.0, 4.0)	(0.0, 3.0)
Missing	1	0	0
PAFAS - parent-child relationship subscale			
Mean (SD)	1.4 (1.4)	1.6 (1.8)	1.4 (1.5)
Range	(0.0, 6.0)	(0.0, 7.0)	(0.0, 6.0)
Missing	1	0	0
Filmed Book-sharing task - sensitivity			
Mean (SD)	3.0 (1.1)	3.0 (1.0)	3.0 (1.1)
Range	(1.0, 5.0)	(1.0, 5.0)	(1.0, 5.0)
Missing	1	3	5
Filmed Book-sharing task - reciprocity			
Mean (SD)	2.7 (1.3)	2.7 (1.2)	2.8 (1.2)
Range	(1.0, 5.0)	(1.0, 5.0)	(1.0, 5.0)
Missing	1	3	5
Filmed Responsive Interactions task - sensitivity			
Mean (SD)	1.9 (0.8)	2.0 (0.8)	2.0 (0.8)
Range	(1.0, 4.0)	(1.0, 4.5)	(1.0, 4.2)
Missing	3	2	5
Filmed Don't Touch and Clean up tasks - mean of total z-scores guidance on two tasks			
Mean (SD)	0.0 (0.7)	-0.0 (0.8)	0.0 (0.8)
Range	(-1.5, 1.6)	(-1.4, 2.2)	(-1.5, 2.3)
Missing	0	1	0
Harsh parenting & Maltreatment			
PAFAS questionnaire - coercive subscale			
Mean (SD)	4.9 (2.4)	4.7 (2.5)	4.8 (2.6)
Range	(0.0, 12.0)	(0.0, 11.0)	(0.0, 12.0)
Missing	1	0	0
Filmed 'Don't touch' and 'Clean Up' Tasks – mean of coercion total z-scores on two tasks			

	ACT (N=123)	DBS (N=124)	Control (N=122)
Mean (SD)	-0.0 (0.7)	0.0 (0.7)	0.0 (0.8)
Range	(-0.7, 2.5)	(-0.7, 3.0)	(-1.1, 3.4)
Missing	0	1	0
Attitudes about physical punishment average score (the higher, the greater endorsement of spanking)			
Mean (SD)	3.0 (0.9)	2.9 (0.8)	3.0 (0.9)
Range	(1.0, 5.0)	(1.0, 5.0)	(1.0, 5.0)
Missing	1	0	0
JVQ Composite Score: Any Maltreatment, n (%)			
Yes	21 (17.1)	20 (16.1)	20 (16.4)
No	101 (82.1)	104 (83.9)	102 (83.6)
Missing	1 (0.8)	-	-
Stress measures			
Maternal Perceived Stress Scale			
Mean (SD)	16.8 (5.7)	16.5 (6.8)	16.4 (7.3)
Range	(0.0, 33.0)	(2.0, 31.0)	(0.0, 37.0)
Missing	1	0	0
Maternal Pelotas Parenting Stress Index			
Mean (SD)	7.6 (3.7)	7.7 (3.4)	8.0 (4.2)
Range	(1.0, 22.0)	(1.0, 19.0)	(1.0, 22.0)
Missing	1	0	0
Maternal cortisol - 3-month levels from hair samples			
Mean (SD)	6.3 (2.8)	6.3 (3.4)	6.3 (2.8)
Range	(1.5, 18.9)	(2.4, 26.0)	(0.2, 18.3)
Missing	2	3	2
Child cortisol - 3-month levels from hair samples			
Mean (SD)	9.3 (5.4)	9.1 (4.4)	9.4 (5.4)
Range	(3.5, 37.4)	(3.0, 28.8)	(2.9, 35.0)
Missing	11	11	2

ACT: Raising Safe Kids Program (ACT); DBS: dialogic book-sharing programme; SD: standard deviation; JVQ: Juvenile Victimization Questionnaire

Tables showing results for the effects of ACT and DBS in the PIÁ trial

Table S11: Unadjusted means for primary outcome of child aggression, and component scores, by intervention and time point

	ACT		DBS		Control	
	**N**	**Mean (SD)**	**N**	**Mean (SD)**	**N**	**Mean (SD)**
**INTENTION-TO-TREAT**	**(n=123)**		**(n=124)**		**(n=122)**	
Baseline						
Child aggression combined score (mean of z-scores)	122	-0.03 (0.9)	124	-0.06 (0.9)	122	0.08 (0.9)
Component: CBCL aggression subscale z-score	122	-0.06 (1.0)	124	-0.04 (1.0)	122	0.10 (1.0)
Component: ELDEQ aggression z-score	119	0.02 (1.1)	124	-0.08 (1.0)	122	0.06 (1.0)
Follow-up (8-months)						
Child aggression combined score (mean of z-scores)	122	-0.39 (0.9)	124	-0.48 (0.8)	122	-0.40 (0.8)
Component: CBCL aggression subscale z-score	122	-0.61 (1.0)	124	-0.59 (0.9)	122	-0.55 (0.8)
Component: ELDEQ aggression z-score	122	-0.17 (1.0)	124	-0.36 (0.9)	122	-0.25 (1.0)
**PER-PROTOCOL**	**(n=79)**		**(n=95)**		**(n=122)**	
Baseline						
Child aggression combined score (mean of z-scores)	78	0.03 (1.0)	95	-0.02 (0.9)	122	0.08 (0.9)
Component: CBCL aggression subscale z-score	78	0.04 (1.0)	95	-0.00 (1.0)	122	0.10 (1.0)
Component: ELDEQ aggression z-score	76	0.05 (1.1)	95	-0.05 (1.0)	122	0.06 (1.0)
Follow-up (8-months)						
Child aggression combined score (mean of z-scores)	79	-0.33 (1.0)	95	-0.47 (0.8)	122	-0.40 (0.8)
Component: CBCL aggression subscale z-score	79	-0.55 (1.0)	95	-0.58 (0.9)	122	-0.55 (0.8)
Component: ELDEQ aggression z-score	79	-0.11 (1.0)	95	-0.36 (0.9)	122	-0.25 (1.0)

ACT: Raising Safe Kids Program; DBS: dialogic book-sharing programme; SD: standard deviation; CBCL: Child Behaviour Checklist; ELDEQ: ELDEQ Study Questionnaire. Z-scores are based on the mean and standard deviation of the outcome at baseline

Table S12: Child aggression outcomes at 8-month follow-up and estimates of intervention effects

	n and adjusted means (95% CI)						Intervention Effect Size	
	ACT		DBS		Control		ACT versus Control	DBS versus Control
**INTENTION-TO-TREAT**	**(N=123)**		**(N=124)**		**(N=122)**		**SMD (95% CI)**	**SMD (95% CI)**
CBCL-ELDEQ combined score (primary outcome)	114	-0.30 (-0.43,-0.17)	115	-0.36 (-0.49,-0.23)	114	-0.41 (-0.54, -0.28)	0.11 (-0.05, 0.27)	0.05 (-0.11, 0.21)
CBCL	114	13.93 (12.77,15.09)	115	14.06 (12.88,15.23)	114	13.59 (12.42, 14.77)	0.05 (-0.15, 0.25)	0.07 (-0.15, 0.28)
ELDEQ	111	5.28 (4.69,5.87)	115	4.86 (4.27,5.45)	114	4.83 (4.24, 5.42)	0.12 (-0.07, 0.31)	0.01 (-0.20, 0.22)
Filmed LabTab	108	0.53 (0.46,0.61)	112	0.51 (0.43,0.58)	110	0.62 (0.54, 0.69)	-0.22 (-0.49, 0.03)	-0.30 (-0.58, -0.03)
Filmed Clean-up and Don't Touch	114	-0.40 (-0.52,-0.28)	114	-0.42 (-0.54,-0.30)	111	-0.38 (-0.50, -0.26)	-0.03 (-0.18, 0.12)	-0.04 (-0.20, 0.11)
Interviewer rating*	114	36 (31.6%)	114	42 (36.8%)	112	48 (42.9%)	OR = 0.63 (0.35, 1.12)	OR = 0.69 (0.39, 1.23)
**PER PROTOCOL**	**(N=79)**		**(N=95)**		**(N=122)**		**SMD (95% CI)**	**SMD (95% CI)**
CBCL-ELDEQ combined score (primary outcome)	73	-0.26 (-0.42,-0.10)	90	-0.34 (-0.49,-0.19)	114	-0.39 (-0.53, -0.26)	0.13 (-0.05, 0.32)	0.06 (-0.12, 0.23)
CBCL	73	14.18 (12.73,15.63)	90	14.21 (12.83,15.59)	114	13.68 (12.47, 14.90)	0.07 (-0.16, 0.29)	0.08 (-0.16, 0.31)
ELDEQ	71	5.53 (4.80,6.26)	90	4.98 (4.29,5.67)	114	4.89 (4.29, 5.49)	0.17 (-0.05, 0.39)	0.03 (-0.21, 0.26)
Filmed LabTab	68	0.55 (0.45,0.64)	88	0.49 (0.40,0.58)	110	0.61 (0.54, 0.69)	-0.19 (-0.49, 0.11)	-0.35 (-0.63, -0.05)
Filmed Clean-up and Don't Touch	73	-0.41 (-0.55,-0.26)	90	-0.44 (-0.58,-0.30)	111	-0.35 (-0.47, -0.22)	-0.06 (-0.23, 0.11)	-0.10 (-0.26, 0.07)
Interviewer rating*	73	24 (32.9%)	90	31 (34.4%)	112	48 (42.9%)	OR = 0.61 (0.31, 1.18)	OR = 0.60 (0.31, 1.14)

ACT: Raising Safe Kids Program; DBS: dialogic book-sharing programme; CI: confidence interval.

Linear regression models adjusted for baseline measure, child's age, child's sex (female/male), maternal education (in years), maternal depression (normal/screening for depression), neighbourhood, child maltreatment (yes/no), Parenting and Family Adjustment Scales (PAFAS) coercive parenting, combined child language, child Go/No-Go task and child callous-unemotional traits.

*Interviewer rated child defiance was modelling using a logistic regression model and is presented as n (%) and odds ratios. The outcome was modelled as any defiance (a bit and a lot) compared to no defiance.

Combined child language score (as covariate) was imputed using multiple imputation by chained equations separately for each population. Imputation models included all variables in the analysis model and previous measures of language measured at 24 months (standardised receptive language and standardised expressive language).

SMD (standardised mean difference) was calculated as the adjusted mean difference where the outcome was a z-score, or dividing the estimate of the adjusted mean difference and confidence limits by the equivalent population standard deviation for outcomes that were not z-scores.

Table S13: Parenting and maltreatment outcomes and estimates of intervention effects

	Months post intervention	n and adjusted means (95% CI)						Intervention Effect Size	
		ACT		DBS		Control		ACT versus Control	DBS versus Control
**INTENTION-TO-TREAT**		(n=123)		(n=124)		(n=122)		SMD (95% CI)	SMD (95% CI)
**Positive Parenting**									
Combined positive parenting score	1	117	0.10 (-0.10, 0.29)	119	0.35 (0.16, 0.54)	117	0.15 (-0.05, 0.35)	-0.05(-0.20,0.10)	0.21(0.06,0.36)
	8	121	0.34 (0.15, 0.53)	123	0.47 (0.29, 0.66)	120	0.29 (0.10, 0.49)	0.05 (-0.09,0.18)	0.18 (0.04,0.32)
PAFAS - positive encouragement	1	120	0.69 (0.40, 0.97)	123	0.60 (0.32, 0.88)	120	0.67 (0.37, 0.96)	0.02 (-0.22,0.26)	-0.08 (-0.34,0.18)
	8	121	0.78 (0.48, 1.07)	124	0.67 (0.38, 0.95)	121	0.76 (0.46, 1.06)	0.02 (-0.21,0.25)	-0.09 (-0.34,0.15)
PAFAS - parent-child relationship	1	120	1.67 (1.15, 2.19)	123	1.43 (0.93, 1.94)	120	1.48 (0.95, 2.02)	0.11 (-0.12,0.34)	-0.03 (-0.26,0.20)
	8	121	1.67 (1.12, 2.23)	124	1.43 (0.89, 1.97)	121	1.51 (0.93, 2.08)	0.08 (-0.15,0.32)	-0.04 (-0.27,0.19)
Filmed Book-sharing sensitivity	1	122	3.12 (2.79, 3.44)	123	3.46 (3.14, 3.77)	122	3.08 (2.75, 3.42)	0.03 (-0.23,0.29)	0.35 (0.09,0.62)
	8	122	3.55 (3.25, 3.85)	123	3.75 (3.46, 4.05)	122	3.46 (3.14, 3.77)	0.10 (-0.14,0.35)	0.33 (0.08,0.57)
Filmed Book-sharing reciprocity	1	122	2.99 (2.62, 3.37)	123	3.43 (3.07, 3.80)	122	3.01 (2.62, 3.40)	-0.01 (-0.27,0.25)	0.37 (0.11,0.63)
	8	122	3.62 (3.25, 4.00)	123	3.86 (3.50, 4.23)	122	3.54 (3.15, 3.93)	0.07 (-0.18,0.32)	0.27 (0.02,0.53)
Filmed Responsive Interactions	1	116	1.69 (1.49, 1.88)	118	1.82 (1.63, 2.01)	113	1.70 (1.49, 1.90)	-0.02 (-0.25,0.22)	0.19 (-0.03,0.40)
	8	120	2.42 (2.21, 2.63)	122	2.62 (2.41, 2.82)	118	2.51 (2.29, 2.73)	-0.12 (-0.37,0.14)	0.14 (-0.09,0.36)
Filmed Clean-up and Don't Touch - guidance	1	117	0.30 (0.04, 0.56)	119	0.49 (0.24, 0.74)	117	0.42 (0.16, 0.68)	-0.12 (-0.33,0.09)	0.07 (-0.14,0.28)
	8	121	-0.05 (-0.29, 0.19)	123	-0.07 (-0.30, 0.17)	119	-0.15 (-0.40, 0.10)	0.10 (-0.08,0.28)	0.08 (-0.10,0.26)
**Harsh parenting & Maltreatment**									
PAFAS - coercive parenting	1	120	4.40 (3.91, 4.89)	123	4.72 (4.25, 5.18)	120	4.81 (4.31, 5.32)	-0.17 (-0.36,0.02)	-0.04 (-0.24,0.16)
	8	121	4.46 (3.98, 4.93)	124	4.51 (4.06, 4.96)	121	4.35 (3.86, 4.84)	0.05 (-0.15,0.24)	0.07 (-0.12,0.26)
	1	117	-0.07 (-0.28, 0.15)	119	0.03 (-0.17, 0.24)	117	0.16 (-0.06, 0.38)	-0.23 (-0.46,-0.01)	-0.13 (-0.35,0.10)

Filmed Clean-up and Don't Touch tasks - coercion	8	121	-0.31 (-0.50, -0.12)	123	-0.25 (-0.43, -0.06)	119	-0.18 (-0.38, 0.02)	-0.13 (-0.31,0.05)	-0.06 (-0.24,0.11)
Attitude favouring spanking	1	120	2.86 (2.69, 3.02)	123	2.98 (2.81, 3.14)	120	3.08 (2.91, 3.24)	-0.24 (-0.45,-0.03)	-0.12 (-0.34,0.10)
JVQ: Any Maltreatment*	8	121	12 (9.9%)	124	4 (3.2%)	122	12 (9.8%)	OR=1.16 (0.44,3.09)	OR=0.23 (0.06,0.81)
**PER PROTOCOL**			**(n=79)**		**(n=95)**		**(n=122)**	**SMD (95% CI)**	**SMD (95% CI)**
**Positive Parenting**									
Combined positive parenting score	1	75	-0.03 (-0.24, 0.18)	93	0.37 (0.18, 0.57)	117	0.13 (-0.07, 0.34)	-0.17 (-0.34,-0.00)	0.24 (0.08,0.40)
	8	78	0.29 (0.08, 0.49)	95	0.50 (0.31, 0.70)	120	0.28 (0.08, 0.48)	0.00 (-0.15,0.16)	0.22 (0.08,0.37)
PAFAS - positive encouragement	1	76	0.63 (0.32, 0.95)	94	0.62 (0.32, 0.92)	120	0.66 (0.35, 0.97)	-0.03 (-0.31,0.25)	-0.04 (-0.31,0.23)
	8	78	0.82 (0.49, 1.14)	95	0.68 (0.37, 0.99)	121	0.75 (0.43, 1.06)	0.07 (-0.20,0.34)	-0.06 (-0.33,0.20)
PAFAS - parent-child relationship	1	76	1.80 (1.21, 2.39)	94	1.45 (0.88, 2.01)	120	1.44 (0.85, 2.02)	0.21 (-0.05,0.46)	0.01 (-0.25,0.26)
	8	78	1.85 (1.21, 2.49)	95	1.47 (0.86, 2.07)	121	1.46 (0.84, 2.08)	0.19 (-0.07,0.45)	0.00 (-0.25,0.25)
Filmed Book-sharing sensitivity	1	78	2.91 (2.56, 3.27)	95	3.52 (3.18, 3.86)	122	3.04 (2.69, 3.39)	-0.11 (-0.41,0.17)	0.45 (0.18,0.73)
	8	78	3.43 (3.10, 3.76)	95	3.80 (3.48, 4.12)	122	3.41 (3.08, 3.74)	0.02 (-0.27,0.31)	0.42 (0.16,0.69)
Filmed Book-sharing reciprocity	1	78	2.73 (2.32, 3.14)	95	3.50 (3.12, 3.89)	122	2.98 (2.57, 3.38)	-0.22 (-0.52,0.08)	0.45 (0.17,0.72)
	8	78	3.61 (3.20, 4.02)	95	3.96 (3.57, 4.35)	122	3.51 (3.11, 3.91)	0.08 (-0.20,0.37)	0.38 (0.11,0.65)
Filmed Responsive Interactions	1	74	1.62 (1.40, 1.84)	92	1.81 (1.60, 2.01)	113	1.67 (1.46, 1.89)	-0.08 (-0.37,0.20)	0.21 (-0.04,0.44)
	8	76	2.48 (2.24, 2.71)	94	2.63 (2.41, 2.85)	118	2.49 (2.26, 2.71)	-0.01 (-0.29,0.28)	0.17 (-0.07,0.41)
Filmed Clean-up and Don't Touch - guidance	1	75	0.24 (-0.04, 0.52)	93	0.50 (0.24, 0.77)	117	0.43 (0.17, 0.70)	-0.20 (-0.44,0.04)	0.07 (-0.16,0.30)
	8	78	-0.13 (-0.39, 0.13)	95	-0.05 (-0.30, 0.20)	119	-0.13 (-0.39, 0.12)	0.00 (-0.19,0.20)	0.08 (-0.10,0.27)
PAFAS - coercive parenting	1	76	4.22 (3.62, 4.82)	94	4.52 (3.97, 5.07)	120	4.69 (4.13, 5.26)	-0.19 (-0.41,0.04)	-0.07 (-0.29,0.15)
	8	78	4.24 (3.67, 4.81)	95	4.27 (3.74, 4.80)	121	4.23 (3.69, 4.78)	0.00 (-0.22,0.23)	0.02 (-0.20,0.23)
	1	75	-0.09 (-0.35, 0.18)	93	0.06 (-0.18, 0.31)	117	0.20 (-0.04, 0.45)	-0.29 (-0.56,-0.02)	-0.14 (-0.39,0.11)

Filmed Clean-up and Don’t Touch tasks - coercion	8	78	-0.27 (-0.50, -0.04)	95	-0.30 (-0.51, -0.08)	119	-0.14 (-0.37, 0.08)	-0.12 (-0.32,0.08)	-0.15 (-0.34,0.04)
Attitude favouring spanking	1	76	2.75 (2.55, 2.95)	94	2.96 (2.78, 3.14)	120	3.07 (2.90, 3.25)	-0.36 (-0.61,-0.12)	-0.13 (-0.38,0.11)
JVQ: Any Maltreatment*	8	78	8 (10.3%)	95	3 (3.2%)	122	12 (9.8%)	OR=1.52 (0.50,4.62)	OR=0.20 (0.05,0.88)

ACT: Raising Safe Kids Program; DBS: dialogic book-sharing programme; CI: confidence interval.

Linear regression models adjusted for baseline measure, child's age, child's sex (female/male), maternal education (in years), maternal depression (normal/screening for depression), neighbourhood and model specific covariates specified in the SAP were fitted. Mixed regression models with neighbourhood as a random effect were fitted where possible.

*JVQ: Composite Score 5 Any Maltreatment was modelled using a logistic regression model and is presented as n (%) and odds ratios. The outcome was collected as yes/no. The model was adjusted for the generic covariates and model specific variables specified in the SAP except for neighbourhood and maternal problem drinking due to model convergence issues.

SMD (standardised mean difference) was calculated as the adjusted mean difference where the outcome was a z-score, or dividing the estimate of the adjusted mean difference and confidence limits by the equivalent population standard deviation for outcomes that were not z-scores.

Table S14: Child development outcomes at 8-month follow-up and estimates of intervention effects

	n and adjusted means (95% CI)						Intervention Effect Size	
	ACT		DBS		Control		ACT versus Control	DBS versus Control
**INTENTION-TO-TREAT**	(n=123)		(n=124)		(n=122)		SMD (95% CI)	SMD (95% CI)
**Child language**								
Combined language score	121	-0.02 (-0.23, 0.20)	123	0.18 (-0.03, 0.40)	121	0.11 (-0.10, 0.33)	-0.13 (-0.29, 0.03)	0.07 (-0.09, 0.22)
Expressive language task	119	47.78 (42.78, 52.79)	119	50.46 (45.28, 55.64)	120	47.61 (42.61, 52.62)	0.01 (-0.17, 0.19)	0.15 (-0.03, 0.34)
Receptive language task	121	23.55 (22.08, 25.02)	123	24.68 (23.19, 26.16)	121	24.90 (23.45, 26.34)	-0.24 (-0.43, -0.05)	-0.04 (-0.24, 0.16)
**Child attention and executive functions**								
SDQ - attention/hyperactivity	121	5.48 (5.06, 5.91)	123	5.00 (4.58, 5.43)	122	5.23 (4.81, 5.66)	0.10 (-0.11, 0.31)	-0.08 (-0.28, 0.11)
Filmed Play Alone task - focus	120	3.02 (2.88, 3.16)	119	2.97 (2.83, 3.11)	117	3.01 (2.87, 3.15)	0.01 (-0.24, 0.25)	-0.06 (-0.34, 0.19)
Interviewer rating - attention	121	1.70 (1.54, 1.86)	123	1.70 (1.53, 1.86)	120	1.76 (1.59, 1.92)	-0.08 (-0.31, 0.16)	-0.08 (-0.32, 0.16)
Card sort task – attention shift	120	2.26 (1.72, 2.79)	123	1.93 (1.40, 2.46)	120	2.26 (1.72, 2.79)	0.00 (-0.26, 0.25)	-0.13 (-0.40, 0.14)
Go/No-Go task – impulse control	110	0.36 (0.32, 0.41)	114	0.37 (0.33, 0.42)	107	0.39 (0.35, 0.44)	-0.16 (-0.41, 0.10)	-0.10 (-0.35, 0.15)
Block design task – spatial reasoning	121	3.73 (3.14, 4.32)	120	3.93 (3.33, 4.53)	118	3.70 (3.10, 4.30)	0.01 (-0.22, 0.24)	0.07 (-0.16, 0.30)
**Child empathy-prosocial behaviour**								
Combined empathy-prosocial score	121	-0.13 (-0.26, -0.01)	123	0.04 (-0.09, 0.16)	122	-0.09 (-0.22, 0.03)	-0.04 (-0.20, 0.12)	0.13 (-0.03, 0.29)
Em-Que - emotion contagion	121	3.22 (2.74, 3.69)	123	3.65 (3.18, 4.12)	122	3.23 (2.76, 3.71)	-0.01 (-0.22, 0.21)	0.18 (-0.04, 0.39)
SDQ - prosocial behaviour score	121	8.43 (8.15, 8.71)	123	8.69 (8.42, 8.97)	122	8.48 (8.20, 8.76)	-0.04 (-0.26, 0.18)	0.14 (-0.09, 0.36)
Filmed help task – empathy/prosocial	114	1.36 (1.08, 1.64)	117	1.32 (1.04, 1.60)	114	1.19 (0.90, 1.47)	0.13 (-0.13, 0.40)	0.10 (-0.16, 0.36)
Puppet task – emotion recognition	106	1.70 (1.48, 1.92)	103	1.98 (1.76, 2.20)	103	1.98 (1.75, 2.21)	-0.29 (-0.56, -0.03)	-0.01 (-0.27, 0.25)
Dictator game - altruism	120	3.02 (2.47, 3.57)	123	3.49 (2.95, 4.04)	121	3.24 (2.69, 3.79)	-0.08 (-0.34, 0.17)	0.09 (-0.16, 0.35)

**Child theory of mind (ToM)**								
Sally-Anne task (poor ToM)	82	21 (25.6%)	84	11 (13.1%)	71	18 (25.4%)	OR = 0.96 (0.42, 2.17)	OR = 0.40 (0.15, 1.05)
**PER-PROTOCOL**		**(n=79)**		**(n=95)**		**(n=122)**	**SMD (95% CI)**	**SMD (95% CI)**
**Child language**								
Combined language score	78	-0.04 (-0.29, 0.21)	95	0.16 (-0.09, 0.42)	121	0.08 (-0.15, 0.31)	-0.12 (-0.31, 0.07)	0.08 (-0.10, 0.26)
Expressive language task	76	47.83 (42.48, 53.18)	91	50.10 (44.72, 55.47)	120	47.18 (42.07, 52.28)	0.03 (-0.18, 0.25)	0.15 (-0.05, 0.35)
Receptive language task	78	23.18 (21.56, 24.79)	95	24.61 (22.97, 26.25)	121	24.65 (23.08, 26.22)	-0.27 (-0.50, -0.03)	-0.01 (-0.24, 0.22)
**Child attention and executive functions**								
SDQ - attention/hyperactivity	78	5.52 (5.00, 6.05)	95	5.21 (4.73, 5.69)	122	5.32 (4.89, 5.76)	0.08 (-0.16, 0.32)	-0.04 (-0.25, 0.17)
Filmed Play Alone task - focus	77	3.06 (2.90, 3.23)	93	2.96 (2.81, 3.12)	117	3.00 (2.86, 3.14)	0.10 (-0.19, 0.38)	-0.06 (-0.35, 0.20)
Interviewer rating - attention	78	1.74 (1.55, 1.94)	95	1.74 (1.56, 1.92)	120	1.78 (1.61, 1.95)	-0.04 (-0.32, 0.23)	-0.05 (-0.30, 0.21)
Card sort task – attention shift	77	2.63 (1.96, 3.30)	95	2.13 (1.51, 2.75)	120	2.30 (1.75, 2.86)	0.13 (-0.17, 0.42)	-0.07 (-0.36, 0.23)
Go/No-Go task – impulse control	69	0.36 (0.31, 0.42)	90	0.37 (0.32, 0.42)	107	0.38 (0.34, 0.43)	-0.10 (-0.36, 0.21)	-0.05 (-0.30, 0.20)
Block design task – spatial reasoning	78	3.84 (3.10, 4.58)	93	3.76 (3.07, 4.45)	118	3.67 (3.04, 4.30)	0.05 (-0.21, 0.32)	0.03 (-0.22, 0.27)
**Child empathy-prosocial behaviour**								
Combined empathy-prosocial score	78	-0.22 (-0.38, -0.06)	95	-0.09 (-0.23, 0.06)	122	-0.15 (-0.28, -0.02)	-0.07 (-0.25, 0.10)	0.06 (-0.11, 0.23)
Em-Que - emotion contagion	78	3.17 (2.63, 3.71)	95	3.40 (2.90, 3.91)	122	3.15 (2.68, 3.61)	0.01 (-0.23, 0.25)	0.11 (-0.12, 0.33)
SDQ - prosocial behaviour score	78	8.19 (7.83, 8.55)	95	8.47 (8.14, 8.79)	122	8.39 (8.09, 8.68)	-0.12 (-0.38, 0.13)	0.05 (-0.20, 0.30)
Filmed help task – empathy/prosocial	73	1.43 (1.09, 1.77)	91	1.21 (0.90, 1.53)	114	1.18 (0.90, 1.47)	0.19 (-0.11, 0.49)	0.02 (-0.27, 0.31)
Puppet task – emotion recognition	66	1.61 (1.34, 1.88)	78	1.99 (1.75, 2.24)	103	2.02 (1.78, 2.26)	-0.43 (-0.73, -0.13)	-0.03 (-0.31, 0.25)
Dictator game - altruism	77	3.03 (2.36, 3.71)	95	3.50 (2.87, 4.13)	121	3.29 (2.73, 3.85)	-0.10 (-0.39, 0.19)	0.08 (-0.20, 0.36)
**Child theory of mind (ToM)**								

Sally-Anne task (poor ToM)	50	8 (16.0%)	61	7 (11.5%)	71	18 (25.4%)	OR = 0.37 (0.12, 1.13)	OR = 0.22 (0.06, 0.79)

ACT: Raising Safe Kids Program; DBS: dialogic book-sharing programme; CI: confidence interval.

Linear regression models adjusted for baseline measure, child's age, child's sex (female/male), maternal education (in years), maternal depression (normal/screening for depression), neighbourhood and model specific covariates specified in the SAP were fitted. Mixed regression models with neighbourhood as a random effect were fitted where possible.

*Sally-Anne task was modelling using a logistic regression model and is presented as n (%) and odds ratios. The outcome was pass/fail. The model was adjusted for the generic covariates and triangle task (categories 0-3) as a proxy baseline measure, child maltreatment (yes/no), Parenting and Family Adjustment Scales (PAFAS) coercive parenting, combined positive parenting, Denham's puppet task, combined child language and older age siblings.

Combined child language score was imputed using multiple imputation by chained equations separately for each population. Imputation models included all variables in the analysis model and previous measures of language measured at 24 months (standardised receptive language and standardised expressive language).

SMD (standardised mean difference) was calculated as the adjusted mean difference where the outcome was a z-score, or dividing the estimate of the adjusted mean difference and confidence limits by the equivalent population standard deviation for outcomes that were not z-scores.

Table S15: Stress outcomes and estimates of intervention effects

	Months post intervention	n and adjusted means (95% CI)						Intervention Effect Size	
			ACT		DBS		Control	ACT versus Control	DBS versus Control
**INTENTION-TO-TREAT**			**(n=123)**		**(n=124)**		**(n=122)**	**SMD (95% CI)**	**SMD (95% CI)**
Perceived Stress Scale - Mother	1	120	16.29 (15.07, 17.51)	123	16.12 (14.94, 17.30)	120	15.83 (14.62, 17.04)	0.06 (-0.15, 0.27)	0.04 (-0.16, 0.24)
Pelotas Parenting Stress Index – Mother	1	120	7.54 (6.84, 8.24)	123	7.72 (7.03, 8.41)	120	7.50 (6.80, 8.20)	0.01 (-0.18, 0.20)	0.06 (-0.14, 0.26)
Mother 3-month cortisol average	8	116	7.69 (6.14, 9.23)	112	7.36 (5.81, 8.91)	110	8.01 (6.51, 9.51)	-0.07 (-0.30, 0.15)	-0.17 (-0.43, 0.09)
Child 3-month cortisol average	8	102	9.06 (7.85, 10.26)	97	9.29 (8.10, 10.48)	111	9.36 (8.22, 10.51)	-0.08 (-0.35, 0.18)	-0.02 (-0.29, 0.25)
**PER PROTOCOL**			**(n=79)**		**(n=95)**		**(n=122)**	**SMD (95% CI)**	**SMD (95% CI)**
Parental Stress Scale score	1	76	16.02 (14.48, 17.56)	94	16.12 (14.73, 17.52)	120	16.02 (14.72, 17.32)	0.00 (-0.23, 0.23)	0.01 (-0.20, 0.23)
Pelotas Parental Stress Index score	1	76	7.40 (6.49, 8.31)	94	7.73 (6.87, 8.59)	120	7.58 (6.74, 8.41)	-0.05 (-0.26, 0.17)	0.04 (-0.17, 0.25)
Mother hair cortisol (pg/mg)	8	74	7.36 (5.83, 8.90)	88	7.14 (5.64, 8.64)	110	8.08 (6.65, 9.51)	-0.18 (-0.44, 0.08)	-0.25 (-0.51, 0.02)
Child 3-month cortisol average	8	66	9.55 (8.12, 10.98)	78	8.97 (7.65, 10.29)	111	9.38 (8.15, 10.61)	0.04 (-0.25, 0.34)	-0.11 (-0.41, 0.18)

ACT: Raising Safe Kids Program; DBS: dialogic book-sharing programme; CI: confidence interval.

Linear regression models adjusted for baseline measure, child's age, child's sex (female/male), maternal education (in years), maternal depression (normal/screening for depression), neighbourhood and model specific covariates specified in the SAP were fitted. Mixed regression models with neighbourhood as a random effect were fitted where possible.

SMD (standardised mean difference) was calculated by dividing the estimate of the adjusted mean difference and confidence limits by the equivalent population standard deviation.

Table S16: Child language outcomes at 8 month follow-up and estimates of intervention effects, using complete cases with valid baseline language data (exploratory, sensitivity analyses)

	n and adjusted mean (95% CI)						Intervention Effect Size	
	ACT		DBS		Control		ACT versus Control	DBS versus Control
**INTENTION-TO-TREAT**	**(n=123)**		**(n=124)**		**(n=122)**		**SMD (95% CI)**	**SMD (95% CI)**
Combined language score	85	-0.05 (-0.34, 0.24)	102	0.12 (-0.16, 0.40)	89	0.11 (-0.18, 0.39)	-0.16 (-0.33, 0.02)	0.02 (-0.16, 0.19)
Expressive language task	81	50.00 (43.77, 56.22)	97	52.73 (46.67, 58.78)	83	50.18 (43.94, 56.42)	-0.01 (-0.21, 0.19)	0.14 (-0.07, 0.34)
Receptive language task	82	22.93 (20.93, 24.92)	100	23.93 (21.99, 25.86)	88	24.51 (22.52, 26.51)	-0.29 (-0.51, -0.06)	-0.11 (-0.34, 0.12)
**PER PROTOCOL**	**(n=79)**		**(n=95)**		**(n=122)**		**SMD (95% CI)**	**SMD (95% CI)**
Combined language score	52	-0.07 (-0.37, 0.23)	76	0.10 (-0.19, 0.38)	89	0.09 (-0.19, 0.37)	-0.16 (-0.37, 0.06)	0.01 (-0.19, 0.21)
Expressive language task	49	48.21 (41.85, 54.57)	71	51.16 (45.15, 57.16)	83	48.51 (42.53, 54.49)	-0.02 (-0.25, 0.22)	0.14 (-0.08, 0.35)
Receptive language task	50	23.27 (21.16, 25.37)	74	24.11 (22.13, 26.09)	88	24.84 (22.87, 26.82)	-0.29 (-0.57, -0.01)	-0.14 (-0.39, 0.12)

ACT: Raising Safe Kids Program; DBS: dialogic book-sharing programme; CI: confidence interval.

Linear regression models adjusted for baseline measure, child's age, child's sex (female/male), maternal education (in years), maternal depression (normal/screening for depression), neighbourhood and model specific covariates specified in the SAP were fitted. Mixed regression models with neighbourhood as a random effect were fitted where possible.

SMD (standardised mean difference) was calculated as the adjusted mean difference where the outcome was a z-score, or dividing the estimate of the adjusted mean difference and confidence limits by the equivalent population standard deviation for outcomes that were not z-scores.

Table S17: Child aggression measures at 8-month follow-up, estimates of intervention effects adjusting for generic covariates only (exploratory, sensitivity analyses)

	n and adjusted mean (95% CI)						Intervention Effect Size	
	ACT		DBS		Control		ACT versus Control	DBS versus Control
**INTENTION-TO-TREAT**	**(n=123)**		**(n=124)**		**(n=122)**		**SMD (95% CI)**	**SMD (95% CI)**
CBCL-ELDEQ combined score (primary outcome)	121	-0.35 (-0.46, -0.24)	124	-0.43 (-0.54, -0.32)	122	-0.45 (-0.56, -0.34)	0.11 (-0.05, 0.26)	0.02 (-0.13, 0.17)
CBCL	121	13.41 (12.40, 14.41)	124	13.38 (12.40, 14.36)	122	12.97 (11.96, 13.97)	0.06 (-0.13, 0.25)	0.06 (-0.14, 0.26)
ELDEQ	118	5.23 (4.72, 5.74)	124	4.59 (4.10, 5.09)	122	4.75 (4.25, 5.25)	0.13 (-0.06, 0.32)	-0.05 (-0.24, 0.16)
Filmed LabTab	115	0.55 (0.49, 0.62)	121	0.50 (0.43, 0.56)	117	0.62 (0.55, 0.68)	-0.17 (-0.41, 0.08)	-0.33 (-0.59, -0.08)
Filmed Clean-up and Don't Touch	121	-0.42 (-0.53, -0.31)	123	-0.42 (-0.53, -0.31)	119	-0.41 (-0.53, -0.30)	-0.02 (-0.25, 0.22)	0.00 (-0.25, 0.23)
Interviewer rating*	121	37 (30.6%)	123	45 (36.6%)	120	51 (42.5%)	OR=0.61 (0.35, 1.06)	OR=0.73 (0.42, 1.26)
**PER PROTOCOL**	**(n=79)**		**(n=95)**		**(n=122)**		**SMD (95% CI)**	**SMD (95% CI)**
CBCL-ELDEQ combined score (primary outcome)	78	-0.30 (-0.44, -0.16)	95	-0.43 (-0.55, -0.31)	122	-0.42 (-0.53, -0.31)	0.12 (-0.05, 0.29)	-0.01 (-0.17, 0.16)
CBCL	78	13.69 (12.45, 14.94)	95	13.51 (12.39, 14.62)	122	13.24 (12.25, 14.24)	0.06 (-0.15, 0.27)	0.04 (-0.18, 0.26)
ELDEQ	76	5.45 (4.80, 6.10)	95	4.55 (3.97, 5.13)	122	4.82 (4.30, 5.34)	0.17 (-0.05, 0.38)	-0.08 (-0.30, 0.14)
Filmed LabTab	73	0.56 (0.47, 0.64)	93	0.47 (0.40, 0.55)	117	0.61 (0.54, 0.68)	-0.14 (-0.44, 0.14)	-0.39 (-0.64, -0.11)
Filmed Clean-up and Don't Touch	78	-0.43 (-0.58, -0.28)	95	-0.43 (-0.57, -0.29)	119	-0.40 (-0.53, -0.28)	-0.05 (-0.30, 0.22)	-0.05 (-0.31, 0.21)
Interviewer rating*	78	25 (32.1%)	95	33 (34.7%)	120	51 (42.5%)	OR=0.58 (0.31, 1.09)	OR=0.65 (0.35, 1.19)

ACT: Raising Safe Kids Program; DBS: dialogic book-sharing programme; CI: confidence interval.

Models adjusted for baseline measure, child's age, child's sex (female/male), maternal education (in years), maternal depression (normal/screening for depression), neighbourhood.

*Interviewer rated child defiance was modelling using a logistic regression model and is presented as n (%) and odds ratios. The outcome was modelled as any defiance (a bit and a lot) compared to no defiance. SMD (standardised mean difference) was calculated as the adjusted mean difference where the outcome was a z-score, or dividing the estimate of the adjusted mean difference and confidence limits by the equivalent population standard deviation for outcomes that were not z-scores.

Table S18: Parenting outcomes for ACT estimates of intervention effects, restricting sample to participants who completed course with a good facilitator (exploratory, sensitivity analyses)

	Months post intervention	n and adjusted mean (95% CI)				Intervention Effect Size
		ACT		Control		ACT versus Control
**PER PROTOCOL**		(n=45)		(n=122)		SMD (95% CI)
**Positive Parenting**						
Combined positive parenting score	1	44	0.04 (-0.20, 0.28)	117	0.19 (-0.02, 0.39)	-0.15 (-0.34, 0.04)
	8	45	0.33 (0.10, 0.56)	120	0.34 (0.13, 0.54)	-0.00 (-0.18, 0.17)
PAFAS - positive encouragement	1	44	0.66 (0.29, 1.03)	120	0.62 (0.31, 0.94)	0.03 (-0.30, 0.38)
	8	45	0.96 (0.57, 1.34)	121	0.71 (0.39, 1.04)	0.23 (-0.09, 0.55)
PAFAS - parent-child relationship	1	44	1.77 (1.08, 2.46)	120	1.40 (0.80, 2.01)	0.22 (-0.10, 0.53)
	8	45	2.17 (1.41, 2.93)	121	1.43 (0.79, 2.07)	0.35 (0.04, 0.66)
Filmed Book-sharing sensitivity	1	45	2.94 (2.53, 3.36)	122	3.12 (2.77, 3.47)	-0.18 (-0.53, 0.18)
	8	45	3.51 (3.13, 3.89)	122	3.49 (3.16, 3.83)	0.02 (-0.32, 0.36)
Filmed Book-sharing reciprocity	1	45	2.80 (2.31, 3.29)	122	3.09 (2.66, 3.51)	-0.25 (-0.61, 0.11)
	8	45	3.72 (3.22, 4.21)	122	3.62 (3.19, 4.04)	0.08 (-0.27, 0.43)
Filmed Responsive Interactions	1	44	1.69 (1.42, 1.95)	113	1.76 (1.53, 1.99)	-0.12 (-0.47, 0.24)
	8	44	2.56 (2.27, 2.85)	118	2.57 (2.33, 2.81)	-0.01 (-0.38, 0.34)
Filmed Clean-up and Don't Touch - guidance	1	44	0.35 (0.02, 0.68)	117	0.47 (0.19, 0.74)	-0.12 (-0.40, 0.16)
	8	45	-0.12 (-0.42, 0.18)	119	-0.10 (-0.36, 0.16)	-0.02 (-0.25, 0.21)
**Harsh Parenting & Maltreatment**						
PAFAS - coercive parenting	1	44	3.99 (3.25, 4.73)	120	4.55 (3.96, 5.15)	-0.22 (-0.47, 0.04)

	8	45	3.92 (3.23, 4.61)	121	4.09 (3.52, 4.66)	-0.08 (-0.34, 0.19)
Filmed Clean-up and Don’t Touch tasks - coercion	1	44	-0.12 (-0.47, 0.22)	117	0.24 (-0.03, 0.51)	-0.36 (-0.69, -0.03)
	8	45	-0.10 (-0.40, 0.19)	119	-0.11 (-0.36, 0.13)	0.01 (-0.24, 0.25)
Attitude favouring spanking	1	44	2.75 (2.50, 2.99)	120	3.08 (2.91, 3.25)	-0.33 (-0.59, -0.07)
JVQ: Any Maltreatment*	8	45	3 (6.7%)	122	12 (9.8%)	OR=0.80 (0.17, 3.67)

ACT: Raising Safe Kids Program; DBS: dialogic book-sharing programme; CI: confidence interval.

Linear regression models adjusted for baseline measure, child's age, child's sex (female/male), maternal education (in years), maternal depression (normal/screening for depression), neighbourhood and model specific covariates specified in the SAP were fitted. Mixed regression models with neighbourhood as a random effect were fitted where possible.

*JVQ Composite Score 5 Any Maltreatment was modelled using a logistic regression model and is presented as n (%) and odds ratios. The outcome was collected as yes/no. The model was adjusted for the generic covariates and model specific variables specified in the SAP except for neighbourhood and maternal problem drinking due to model convergence issues.

SMD (standardised mean difference) was calculated as the adjusted mean difference where the outcome was a z-score, or dividing the estimate of the adjusted mean difference and confidence limits by the equivalent population standard deviation for outcomes that were not z-scores.

Table S19: Moderator analyses (intention-to-treat population)

Outcome	Months post intervention	Moderator (measured at baseline or pre-baseline)	ACT versus Control		DBS verses Control	
			N	p-value‡	N	p-value‡
**CBCL-ELDEQ combined score (primary outcome)***	8	Baseline measure	228	0.446	229	0.308
	8	Child sex	228	0.194	229	0.122
	8	Child age	228	0.404	229	0.622
	8	Child callous-unemotional traits	228	0.36	229	0.744
	8	Maternal education	228	0.278	229	0.481
	8	Maternal depression	228	0.525	229	0.083
	8	Intimate partner violence	228	0.191	229	0.341
	8	PAFAS coercive parenting	228	0.231	229	0.077
	8	Participation in PIM programme	228	0.074	229	0.375
**Combined language score***	8	Baseline measure	237	0.129	244	0.250
	8	Child sex	237	0.303	244	0.126
	8	Child age	237	0.187	244	0.162
	8	Maternal education	237	0.226	244	0.186
	8	Maternal depression	237	0.101	244	0.246
	8	Intimate partner violence	237	0.353	244	0.285
	8	Filmed book-sharing task – sensitivity	…	…	237	0.161
**SDQ - attention/hyperactivity**	8	Baseline measure	243	0.282	245	0.082
	8	Child sex	243	0.295	245	0.117
	8	Child age	243	0.626	245	0.431

Outcome	Months post intervention	Moderator (measured at baseline or pre-baseline)	ACT versus Control		DBS verses Control	
			N	p-value‡	N	p-value‡
	8	Maternal education	243	0.926	245	0.503
	8	Maternal depression	243	0.677	245	0.363
	8	Intimate partner violence	243	0.939	245	0.400
	8	Filmed book-sharing task – sensitivity	…	…	238	0.979
**Block design task – spatial reasoning**	8	Baseline measure	239	0.687	238	0.425
	8	Child sex	239	0.836	238	0.672
	8	Child age	239	0.273	238	0.851
	8	Maternal education	239	0.254	238	0.849
	8	Maternal depression	239	0.249	238	0.212
	8	Intimate partner violence	239	0.514	238	0.435
**Combined empathy-prosocial score**	8	Baseline measure	243	0.161	245	0.590
	8	Child sex	243	0.454	245	0.187
	8	Child age	243	0.601	245	0.890
	8	Maternal education	243	0.700	245	0.687
	8	Maternal depression	243	0.452	245	0.797
	8	Intimate partner violence	243	0.801	245	0.234
**Combined positive parenting score**	1	Baseline measure	234	0.827	236	0.441
	1	Child sex	234	0.973	236	0.855
	1	Child age	234	0.748	236	0.223
	1	Maternal education	234	0.764	236	0.587

Outcome	Months post intervention	Moderator (measured at baseline or pre-baseline)	ACT versus Control		DBS verses Control	
			N	p-value[‡]	N	p-value[‡]
	1	Maternal depression	234	0.708	236	0.890
	8	Baseline measure	241	0.909	243	0.530
	8	Child sex	241	0.989	243	0.502
	8	Child age	241	0.869	243	0.817
	8	Maternal education	241	0.765	243	0.356
	8	Maternal depression	241	0.031	243	0.798
**PAFAS - coercive parenting**	1	Baseline measure	240	0.905	…	…
	1	Child sex	240	0.836	…	…
	1	Child age	240	0.392	…	…
	1	Maternal education	240	0.535	…	…
	1	Maternal depression	240	0.761	…	…
	1	Intimate partner violence	240	0.147	…	…
	8	Baseline measure	242	0.625	…	…
	8	Child sex	242	0.718	…	…
	8	Child age	242	0.710	…	…
	8	Maternal education	242	0.146	…	…
	8	Maternal depression	242	0.524	…	…
	8	Intimate partner violence	242	0.282	…	…
**JVQ: Any Maltreatment**	8	Baseline measure	243	0.775	…	…
	8	Child sex	243	0.461	…	…

Outcome	Months post intervention	Moderator (measured at baseline or pre-baseline)	ACT versus Control		DBS verses Control	
			N	p-value‡	N	p-value‡
	8	Child age	243	0.037	…	…
	8	Maternal education	243	0.524	…	…
	8	Maternal depression	243	0.324	…	…
	8	Intimate partner violence	243	0.440	…	…
**Mother 3-month cortisol average**	8	Baseline measure	226	0.204	222	0.991
	8	Maternal education	226	0.360	222	0.158
	8	Maternal depression	226	0.440	222	0.530
	8	Intimate partner violence	226	0.292	222	0.178
**Child 3-month cortisol average**	8	Baseline measure	213	0.769	208	0.006
	8	Child sex	213	0.176	208	0.700
	8	Child age	213	0.115	208	0.482
	8	Maternal education	213	0.249	208	0.115
	8	Maternal depression	213	0.891	208	0.995
	8	Intimate partner violence	213	0.921	208	0.307

‡ Type 3 p-values for interactions between moderator and intervention (pooled over models from multiply imputed data). Models are adjusted for the same covariates as the corresponding main analyses with the addition of the interaction between moderator and intervention

Additional Information about Maternal Use of ACT and DBS Techniques Post-Intervention

One potential reason why ACT and DBS programmes did not benefit child outcomes might have been if the parenting techniques taught by the programmes were not used by parents, or if those techniques do not associate with child outcomes. In the main trial analyses (Figure 3 and Table S13) some effects of ACT and DBS on parenting techniques were found post-intervention — i.e. parents in the ACT and DBS groups were using relevant parenting techniques more than parents in the control group. In exploratory analyses, we additionally considered the following data concerning parental use of ACT and DBS parenting techniques.

First, we examined maternal reported intentions to continue using the techniques taught in the programmes, at post-intervention assessment. Among parents in the ACT group who responded (N=100), 99% planned to keep using the tools learnt in ACT with their child, and 100% of parents in DBS group who responded (N=104) planned to keep book sharing with their child. Second, we measured the time mothers actually spent book-sharing in the month post-intervention. Most mothers self-reported book-sharing with their child several times per week, for at least five minutes (Table S20). Third, we examined correlations between ACT and DBS techniques post intervention and two key child outcomes at follow-up (child aggression as the trial primary outcome, and child language – as the focal child outcome of DBS). These results (Table S21) show that ACT and DBS parenting techniques (less coercion, more book-sharing and more high-quality book-sharing) do associate with relevant child outcomes.

Table S20: Descriptives of time implementing book-sharing in DBS group at one month post-intervention (N = 124)

**Booksharing past month**	**N (%)**
**Times per week**	
None	1 (1.0)
1 time	15 (14.4)
2-3 times	44 (42.3)
4-5 times	22 (21.2)
6-7 times	22 (21.2)
Missing*	20
**Minutes each time**	
<5 mins	4 (3.9)
Around 5 mins	9 (8.7)
Between 5-10 mins	19 (18.5)
Between 10-15 mins	31 (30.1)
> 15 mins	40 (38.8)
Missing*	21

Note. For the DBS adherence group (n = 95), 1 case was missing data. For the non-adherence group (n=29), 19 did not respond to the question on number of times book-shared per week, and 20 did not respond to the question on minutes each time. Note these data are not available for ACT or Control groups.

Table S21: Correlations between key measures of ACT and DBS parenting techniques (post-intervention) and child outcomes (follow-up)

Parenting techniques	Child Outcomes	
	Aggression (primary outcome)	Language
**Coercive discipline PAFAS**	.44, p<.001	-.05, p=.354
**Coercive discipline filmed**	.05, p=.317	-.10, p=.051
**Booksharing time**	-.06, p=.057	.21, p=.031
**Booksharing sensitivity**	-.01, p=.794	.26, p<.001
**Booksharing reciprocity**	.00, p=.956	.30, p<.001

Note. Analyses run for whole trial sample (N=369) except for Booksharing time, which is only available for DBS group (N=124). Booksharing time = multiplicative of times per week * minutes each time (Table S20). Child language is the combined expressive and receptive language score at follow-up.

Details of Processing of Hair for Cortisol Measurement

Hair samples provide a measure of cortisol concentration average over a three-month period and represent a useful measure of chronic levels of cortisol (and thus "toxic stress"), unlike other types of samples, such as saliva, blood, or urine which are used to measure acute cortisol concentrations at the time of collection. Hair cortisol was measured using a standardized protocol of hair cutting and storage, washing, grinding, hormone extraction and quantification. After hair cutting, the entire process was conducted at the laboratory of the Postgraduate Program in Epidemiology, Federal University of Pelotas. To cut the hair, a trained fieldworker sanitized the scissors and dried the hair of the participant with a hair dryer if it was wet or dump. Then, the fieldworker identified the posterior vertex of the head and tied approximately 100 hair strands with a string, placed the scissors as near as possible to the scalp, and cut the hair. The hair sample was then stored inside a Bristol board with identification of the hair that came closest to the scalp, and these materials were sealed inside a Ziploc bag. Whenever the hair was too short at the vertex, the hair was collected from the tuft; if the hair was too short at the tuft as well, then several hair samples from the head was collected to obtain enough hair strands for analysis.

The laboratory technicians measured 3 centimetres of the hair cut closest to the scalp to obtain the sample to be analyzed. The sample was placed into a Falcon 50 ml Conical Centrifuge Tube, washed with 12 ml of isopropanol, and shook by hand for 2 minutes (after which the isopropanol was discarded) - this procedure was performed twice. When completely dry, the samples were ground using a mill (Retsch NM400). For cortisol extraction, around 35 mg of ground hair powder plus 1ml of absolute ethanol was shaken for 48 hours (Orbit LS Labnet) and then vortexed. Then, the sample was rotated in the centrifuge (Sigma) at 12.000 rpm, and the supernatant was extracted and kept in a separate tube. Another 1ml of absolute ethanol was added to the original tube and, after a further 48 hours of shaking, it was vortexed, and the supernatant was transferred for the tube containing the first-round supernatant. Those tubes were left drying for 5 days, until completely dry. Samples were suspended in 150 µl of assay diluent for 24h and then assayed in duplicate by ELISA (Enzyme-linked immunosorbentassay) using the High Sensitivity Salivary Cortisol Immunoassay Kit (Cat# 1-3002, Salimetrics, Pennsylvania), as per the manufacturer's instructions. Cortisol levels were measured using an ELISA plate reader (Spectramax 190) and were expressed in pg/mg. Intra- and interassay coefficients of variance were below 10% in the present study.

References for Supplement

Abell, F., Happé, F., & Frith, U. (2000). Do triangles play tricks? Attribution of mental states to animated shapes in normal and abnormal development. *Cognitive Development, 15*(1), 1-16. doi:https://doi.org/10.1016/S0885-2014(00)00014-9

Abidin, R. R. (1997). Parenting Stress Index: A measure of the parent–child system. In *Evaluating stress: A book of resources.* (pp. 277-291). Lanham, MD, US: Scarecrow Education.

Achenbach, T. M., & Rescorla, L. A. (2000). *Manual for the ASEBA Preschool Forms and Profiles*. Burlington, VT: Research Center for Children, Youth, and Families, University of Vermont.

Baron-Cohen, S., Leslie, A. M., & Frith, U. (1985). Does the autistic child have a "theory of mind" ? *Cognition, 21*(1), 37-46. doi:https://doi.org/10.1016/0010-0277(85)90022-8

Benenson, J. F., Pascoe, J., & Radmore, N. (2007). Children's altruistic behavior in the dictator game. *Evolution and Human Behavior, 28*(3), 168-175. doi:10.1016/j.evolhumbehav.2006.10.003

Buttelmann, D., Carpenter, M., & Tomasello, M. (2009). Eighteen-month-old infants show false belief understanding in an active helping paradigm. *Cognition, 112*(2), 337-342. doi:https://doi.org/10.1016/j.cognition.2009.05.006

Capovilla, F. C., Negrão, M. D., & Damázio, M. (2011). *Teste de Vocabulário Auditivo e Teste de Vocabulário Expressivo [Test of Receptive and Expressive Vocabulary]*. Retrieved from Brazil:

Caspi, A., & Silva, P. A. (1995). Temperamental Qualities at Age Three Predict Personality Traits in Young Adulthood: Longitudinal Evidence from a Birth Cohort. *Child Development, 66*(2), 486-498. Retrieved from <Go to ISI>://A1995QX04000016

Cooper, P. J., Vally, Z., Cooper, H., Radford, T., Sharples, A., Tomlinson, M., & Murray, L. (2014). Promoting Mother–Infant Book Sharing and Infant Attention and Language Development in an Impoverished South African Population: A Pilot Study. *Early Childhood Education Journal, 42*(2), 143-152. doi:10.1007/s10643-013-0591-8

Côté, S. M., Boivin, M., Nagin, D. S., & et al. (2007). The role of maternal education and nonmaternal care services in the prevention of children's physical aggression problems. *Archives of General Psychiatry, 64*(11), 1305-1312. doi:10.1001/archpsyc.64.11.1305

Deater-Deckard, K., Lansford, J. E., Dodge, K. A., Pettit, G. S., & Bates, J. E. (2003). The Development of Attitudes About Physical Punishment: An 8-Year Longitudinal Study. *Journal of family psychology : JFP : journal of the Division of Family Psychology of the American Psychological Association (Division 43), 17*(3), 351-360. doi:10.1037/0893-3200.17.3.351

Denham, S. A., Bassett, H. H., Way, E., Kalb, S., Warren-Khot, H., & Zinsser, K. (2014). "How Would You Feel? What Would You Do?" Development and Underpinnings of Preschoolers' Social Information Processing. *Journal of research in childhood education : JRCE / Association for Childhood Education International, 28*(2), 182-202. doi:10.1080/02568543.2014.883558

Devine, R. T., & Hughes, C. (2018). Family Correlates of False Belief Understanding in Early Childhood: A Meta-Analysis. *Child Development, 89*(3), 971-987. doi:10.1111/cdev.12682

Dowdall, N., Melendez-Torres, G. J., Murray, L., Gardner, F., Hartford, L., & Cooper, P. J. (2020). Shared Picture Book Reading Interventions for Child Language Development: A Systematic Review and Meta-Analysis. *Child Development, 91*(2), e383-e399. doi:10.1111/cdev.13225

Ezpeleta, L., de la Osa, N., Granero, R., Penelo, E., & Domènech, J. M. (2013). Inventory of callous-unemotional traits in a community sample of preschoolers. *J Clin Child Adolesc Psychol, 42*(1), 91-105. doi:10.1080/15374416.2012.734221

Finkelhor, D., Hamby, S. L., Ormrod, R., & Turner, H. (2005). The Juvenile Victimization Questionnaire: Reliability, validity, and national norms. *Child Abuse and Neglect, 29*(4), 383-412. doi:https://doi.org/10.1016/j.chiabu.2004.11.001

Finkelhor, D., Hamby, S. L., Turner, H., & Ormrod, R. (2011). *The Juvenile Victimization Questionnaire: 2nd Revision (JVQ-R2)*. Retrieved from Durham, NH:

Gagne, J. (2019). Laboratory Temperament Assessment Battery (Lab-TAB) Retrieved from http://www.uta.edu/faculty/jgagne/labtab/

Garcia-Moreno, C., Jansen, H. A., Ellsberg, M., Heise, L., & Watts, C. H. (2006). Prevalence of intimate partner violence: findings from the WHO multi-country study on women's health and domestic violence. *Lancet, 368*(9543), 1260-1269. doi:10.1016/s0140-6736(06)69523-8

Goodman, R. (2001). Psychometric Properties of the Strengths and Difficulties Questionnaire. *Journal of the American Academy of Child and Adolescent Psychiatry, 40*(11), 1337-1345. doi:10.1097/00004583-200111000-00015

Howard, S. J., & Melhuish, E. (2017). An Early Years Toolbox for Assessing Early Executive Function, Language, Self-Regulation, and Social Development: Validity, Reliability, and Preliminary Norms. *J Psychoeduc Assess, 35*(3), 255-275. doi:10.1177/0734282916633009

Kannass, K. N., Oakes, L. M., & Shaddy, D. J. (2006). A Longitudinal Investigation of the Development of Attention and Distractibility. *Journal of Cognition and Development, 7*(3), 381-409. doi:10.1207/s15327647jcd0703_8

Kochanska, G., & Aksan, N. (1995). Mother-Child Mutually Positive Affect, the Quality of Child Compliance to Requests and Prohibitions, and Maternal Control as Correlates of Early Internalization. *Child Development, 66*(1), 236-254. doi:10.1111/j.1467-8624.1995.tb00868.x

LoBue, V., & Thrasher, C. (2015). The Child Affective Facial Expression (CAFE) set: validity and reliability from untrained adults. *Frontiers in Psychology, 5*. doi:10.3389/fpsyg.2014.01532

Luft, C. D. B., Sanches, S. d. O., Mazo, G. Z., & Andrade, A. (2007). Versão brasileira da Escala de Estresse Percebido: tradução e validação para idosos. *Revista de Saúde Pública, 41*, 606-615. Retrieved from http://www.scielo.br/scielo.php?script=sci_arttext&pid=S0034-89102007000400015&nrm=iso

Martins, R. C., Machado, A. K. F., Shenderovich, Y., Soares, T. B., da Cruz, S. H., Altafim, E. R. P., . . . Murray, J. (2020). Parental attendance in two early-childhood training programmes to improve nurturing care: A randomized controlled trial. *Children and Youth Services Review, 118*, 105418. doi:https://doi.org/10.1016/j.childyouth.2020.105418

Murray, L., De Pascalis, L., Tomlinson, M., Vally, Z., Dadomo, H., MacLachlan, B., . . . Cooper, P. J. (2016). Randomized controlled trial of a book-sharing intervention in a deprived South African community: effects on carer–infant interactions, and their relation to infant cognitive and socioemotional outcome. *Journal of Child Psychology and Psychiatry, 57*(12), 1370-1379. doi:10.1111/jcpp.12605

NICHD. (1998). Early Child Care and Self-Control, Compliance, and Problem Behavior at Twenty-Four and Thirty-Six Months. *Child Development, 69*(4), 1145-1170. doi:https://doi.org/10.1111/j.1467-8624.1998.tb06165.x

Pereira, M., Negrão, M., Soares, I., & Mesman, J. (2014). DECREASING HARSH DISCIPLINE IN MOTHERS AT RISK FOR MALTREATMENT: A RANDOMIZED CONTROL TRIAL. *Infant Mental Health Journal, 35*(6), 604-613. doi:doi:10.1002/imhj.21464

Prime, H., Browne, D., Akbari, E., Wade, M., Madigan, S., & Jenkins, J. M. (2015). The development of a measure of maternal cognitive sensitivity appropriate for use in primary care health settings. *Journal of Child Psychology and Psychiatry, 56*(4), 488-495. doi:10.1111/jcpp.12322

Rieffe, C., Ketelaar, L., & Wiefferink, C. H. (2010). Assessing empathy in young children: Construction and validation of an Empathy Questionnaire (EmQue). *Personality and Individual Differences, 49*(5), 362-367. doi:https://doi.org/10.1016/j.paid.2010.03.046

Sanders, M. R., Morawska, A., Haslam, D. M., Filus, A., & Fletcher, R. (2014). Parenting and Family Adjustment Scales (PAFAS): Validation of a Brief Parent-Report Measure for Use in Assessment of Parenting Skills and Family Relationships. *Child Psychiatry and Human Development, 45*(3), 255-272. doi:10.1007/s10578-013-0397-3

Santos, I. S., Matijasevich, A., Tavares, B. F., Barros, A. J., Botelho, I. P., Lapolli, C., . . . Barros, F. C. (2007). Validation of the Edinburgh Postnatal Depression Scale (EPDS) in a sample of mothers from the 2004 Pelotas Birth Cohort Study. *Cadernos de Saude Publica, 23*(11), 2577-2588. doi:10.1590/s0102-311x2007001100005

Schneider, A., Rodrigues, M., Falenchuk, O., Munhoz, T. N., Barros, A. J. D., Murray, J., . . . Jenkins, J. M. (2021). Cross-Cultural Adaptation and Validation of the Brazilian Portuguese Version of an Observational Measure for Parent–Child Responsive Caregiving. *International Journal of Environmental Research and Public Health, 18*(3), 1246. Retrieved from https://www.mdpi.com/1660-4601/18/3/1246

Taumoepeau, M. (2016). Maternal Expansions of Child Language Relate to Growth in Children's Vocabulary. *Language Learning and Development, 12*(4), 429-446. doi:10.1080/15475441.2016.1158112

Vally, Z., Murray, L., Tomlinson, M., & Cooper, P. J. (2015). The impact of dialogic book-sharing training on infant language and attention: a randomized controlled trial in a deprived South African community. *Journal of Child Psychology and Psychiatry, 56*(8), 865-873. doi:10.1111/jcpp.12352

Wechsler, D., Golombok, S., & Rust, J. (1992). *Weschler Intelligence Scale for Children (Third Edition)*. The Psychological Corporation: London.

Whitehurst, G. J., Falco, F. L., Lonigan, C. J., Fischel, J. E., DeBaryshe, B. D., Valdez-Menchaca, M. C., & Caulfield, M. (1988). Accelerating language development through picture book reading. *Developmental Psychology, 24*(4), 552-559. doi:10.1037/0012-1649.24.4.552

World Health Organization. (2001). AUDIT: the Alcohol Use Disorders Identification Test : guidelines for use in primary health care / Thomas F. Babor ... [et al.]. In (2nd ed ed.). Geneva: World Health Organization.

Appendix A: CONSORT Checklist for the PIA trial

Section/Topic	Item No	Checklist item	Reported at location
**Title and abstract**			
	1a	Identification as a randomised trial in the title	Title
	1b	Structured summary of trial design, methods, results, and conclusions (for specific guidance see CONSORT for abstracts)	Abstract
**Introduction**			
Background and objectives	2a	Scientific background and explanation of rationale	Introduction
	2b	Specific objectives or hypotheses	Introduction
**Methods**			
Trial design	3a	Description of trial design (such as parallel, factorial) including allocation ratio	Methods
	3b	Important changes to methods after trial commencement (such as eligibility criteria), with reasons	N/A
Participants	4a	Eligibility criteria for participants	Methods-Participants
	4b	Settings and locations where the data were collected	Methods-Participants
Interventions	5	The interventions for each group with sufficient details to allow replication, including how and when they were actually administered	Methods-Interventions Supplement Pages 2-7
Outcomes	6a	Completely defined pre-specified primary and secondary outcome measures, including how and when they were assessed	Methods-Assessments Supplement Tables S3-S6
	6b	Any changes to trial outcomes after the trial commenced, with reasons	Methods-Changes to protocol
Sample size	7a	How sample size was determined	Methods-Statistical Analyses
	7b	When applicable, explanation of any interim analyses and stopping guidelines	N/A
Randomisation:			
Sequence generation	8a	Method used to generate the random allocation sequence	Methods-Randomisation
	8b	Type of randomisation; details of any restriction (such as blocking and block size)	Methods-Randomisation
Allocation concealment mechanism	9	Mechanism used to implement the random allocation sequence (such as sequentially numbered containers), describing any steps taken to conceal the sequence until interventions were assigned	Methods-Randomisation
Implementation	10	Who generated the random allocation sequence, who enrolled participants, and who assigned participants to interventions	Methods-Randomisation
Blinding	11a	If done, who was blinded after assignment to interventions (for example, participants, care providers, those assessing outcomes) and how	Methods-Randomisation
	11b	If relevant, description of the similarity of interventions	Methods-Interventions Supplement Pages 2-7
Statistical methods	12a	Statistical methods used to compare groups for primary and secondary outcomes	Methods-Statistical Analyses
	12b	Methods for additional analyses, such as subgroup analyses and adjusted analyses	Methods-Statistical Analyses

**Results**			
Participant flow (a diagram is strongly recommended)	13a	For each group, the numbers of participants who were randomly assigned, received intended treatment, and were analysed for the primary outcome	Figure 1
	13b	For each group, losses and exclusions after randomisation, together with reasons	Figure 1
Recruitment	14a	Dates defining the periods of recruitment and follow-up	Results
	14b	Why the trial ended or was stopped	N/A
Baseline data	15	A table showing baseline demographic and clinical characteristics for each group	Table 1<br>Supplement Tables S9-S10
Numbers analysed	16	For each group, number of participants (denominator) included in each analysis and whether the analysis was by original assigned groups	Supplement Tables S11-S19
Outcomes and estimation	17a	For each primary and secondary outcome, results for each group, and the estimated effect size and its precision (such as 95% confidence interval)	Supplement Tables S11-S19
	17b	For binary outcomes, presentation of both absolute and relative effect sizes is recommended	% and ORs in Supplement Tables S11-S18
Ancillary analyses	18	Results of any other analyses performed, including subgroup analyses and adjusted analyses, distinguishing pre-specified from exploratory	Supplement Tables S16-S18
Harms	19	All important harms or unintended effects in each group (for specific guidance see CONSORT for harms)	Results and Discussion
**Discussion**			
Limitations	20	Trial limitations, addressing sources of potential bias, imprecision, and, if relevant, multiplicity of analyses	Discussion-Limitations paragraph
Generalisability	21	Generalisability (external validity, applicability) of the trial findings	Discussion-Limitations paragraph
Interpretation	22	Interpretation consistent with results, balancing benefits and harms, and considering other relevant evidence	Discussion
**Other information**			
Registration	23	Registration number and name of trial registry	Methods
Protocol	24	Where the full trial protocol can be accessed, if available	Methods
Funding	25	Sources of funding and other support (such as supply of drugs), role of funders	Acknowledgements

*We strongly recommend reading this statement in conjunction with the CONSORT 2010 Explanation and Elaboration for important clarifications on all the items. If relevant, we also recommend reading CONSORT extensions for cluster randomised trials, non-inferiority and equivalence trials, non-pharmacological treatments, herbal interventions, and pragmatic trials. Additional extensions are forthcoming: for those and for up to date references relevant to this checklist, see www.consort-statement.org

Appendix B: Pre-Specified Statistical Analysis Plan

First published at www.doveresearch.org December 2020

Statistical Analysis Plan for the Pelotas Parenting Interventions for Aggression (PIA) trial

Trial registration: RBR-2kwfsk at the Brazilian Ministry of Health Register of Clinical Trials (http://www.ensaiosclinicos.gov.br/)

Effective Date: 3 December 2020

Author: Merryn Voysey, University of Oxford

Reviewers/Approvers:

Joseph Murray, Federal University of Pelotas

Rafaela Martins, Federal University of Pelotas

Signatures:

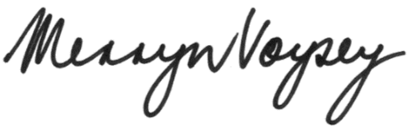

3 December 2020

Merryn Voysey **Date**

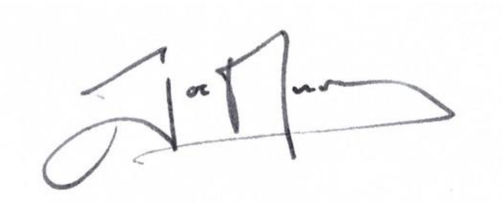

3 December 2020

Joseph Murray **Date**

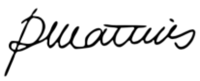

3 December 2020

Rafaela Martins **Date**

Version History

Version	Date	Details
1	3 December 2020	Approved version 1
2	5 January 2024	Approved version 1 with corrected variable name (p3sdqhiperac) shown in red on page 6

Table of Contents

Statistical Analysis Plan for the Pelotas Parenting Interventions for Aggression (PIA) trial 1

1 Introduction: 4

2 Hypotheses: 4

2.1 Primary Hypothesis 4

2.2 Secondary Hypotheses 4

3 Trial Methods: 4

3.1 Trial design 4

3.2 Randomisation 4

3.3 Sample size 5

3.4 Blinding 5

3.5 Interim analysis 5

3.6 Final analysis 5

3.7 Outcome assessments and other measures 5

Table 1. Study Outcomes and Measures 6

4 Statistical principles 9

4.1 Significance level 9

4.2 Multiple comparisons 9

4.3 Analysis Populations 10

4.4 Outliers 10

5 Trial population 10

6 Efficacy Analysis 10

6.1 General analysis considerations 10

6.1.1 Descriptive statistics 10

6.1.2 Missing data 11

6.2 Primary analyses 11

6.3 Sensitivity analyses 11

Table 2. Study Covariates for each Outcome 12

6.4 Mediator effects 15

6.5 Moderator effects 15

Table 3. Mediators and Moderators for each Outcome 16

7 Changes from protocol defined statistical analysis 19

8 References 20

Introduction:

The study aims are to determine, via a three-arm randomised controlled trial, the impact of two group-based, parent training programmes implemented with mothers with young children. The two training programmes are *ACT: Raising Safe Kids*, which primarily aims to reduce harsh discipline and maltreatment against children, and a *Dialogic Book Sharing* (DBS) programme, which primarily aims to stimulate positive parent child interaction and child cognitive development. In particular, we aim to determine the impact of each training programme on child aggressive behaviour (primary outcome), and child cognitive development (secondary outcomes), and parenting practices (secondary outcomes).

Hypotheses:

Primary Hypothesis

1. Compared to a control group of children whose carers receive no additional intervention, children whose carers receive ACT or DBS will evidence significantly less aggressive behaviour.

Secondary Hypotheses

1. Compared to children of families who receive no intervention (the control group), the children in families receiving Dialogic Book-Sharing (DBS) will show less aggression at follow-up, and they will perform better on measures of language, executive function, attention, and empathy/emotion understanding; but parents will show more positive parenting, but parents will not show less harsh and abusive parenting.
2. Compared to children of control group families, the children of families receiving ACT will show less aggression at follow-up, and their parents will show less harsh and abusive parenting and less favourable attitudes about corporal punishment; but the children will not perform better on measures of language, executive function, attention, and empathy/emotion understanding.
3. For both the DBS and ACT groups, children and parents will show less stress at follow-up, and parents will show more positive parenting.

Trial Methods:

Trial design

The study is a three-arm randomized controlled trial (RCT) nested within an ongoing birth cohort study. The 2015 Pelotas Birth Cohort Study, has followed 4,275 children from birth. The cohort includes the entire population of children born in the city of Pelotas, southern Brazil, born in the calendar year 2015. Based on data collected on the cohort when children were aged 24-months, a sub-sample of mother-child pairs were recruited for the PIÁ trial, when children were between 2-3 years old. Interventions were delivered by the Pelotas municipal government staff in local educational facilities, under the supervision of the research team. Baseline, 4-week post-intervention, and 8-month follow-up assessments were conducted for the PIÁ trial.

Randomisation

Randomisation of mother-child pairs to control and the two intervention (ACT and DBS) conditions, was undertaken at the research centre immediately after baseline assessment, minimising for the following dichotomous variables: age of child (<3 years and ≥3 years), child sex (male and female), child aggression score at age 2 years (<4 and ≥4) harsh parental discipline score at age 2 years (<6 and ≥6). The probability that individuals entered into each of the three arms of the trial was 33.3%.

Sample size

The sample comprises mothers from the 2015 Pelotas Birth Cohort Study with children 2-3 years old, who were in the poorest 30% of the population, and who reported that their children showed average-high levels of child aggression at age 24-months.

Power calculation: With alpha set at 0.025 due to two pair-wise comparisons (i.e. between DBS and control, and between ACT and control), and beta at 0.20, each of the three arms in the trial requires a minimum of 104 participants (allowing for 10% attrition to the follow-up assessment) to detect a mid-range effect size of d = 0.45. Therefore a minimum of 312 participants were needed for the study. The actual number of participants recruited to the study was 369 with 369 (100%) followed at post-intervention, and 368 (99.7%) followed-up at 8 months post-intervention.

Blinding

To prevent assessment bias, assessments of children and caregivers have been carried out blind to group allocation, including explicitly asking participants not to reveal their allocation to the data collectors. All coding of video material has been made blind to allocation. The statistician will be provided with individual intervention allocations at the time of database lock, in the form of a categorical variable with three values (1= A, 2 = B, 3 = C) remaining blind to which of A, B, and C refers to the ACT group, the DBS, and the Control group. The statistician will then compare A-B, B-C, and C-A, in order to generate trial results comparing ACT-control and DBS-Control, while remaining blind to intervention status. After primary intention to treat analyses have been completed comparing outcomes in this way, the statistician will be unblinded in order to complete additional per protocol analyses and mediation and moderation analyses comparing ACT-Control, DBS-Control.

Interim analysis

There are no planned interim analyses.

Final analysis

The final analyses will be performed after all subjects have completed their follow-up visit, the data are coded, cleaned and the database locked. At the time of database lock the statistician will request to receive the individual level intervention information from the trial manager.

Outcome assessments and other measures

Table 1 shows the trial outcome constructs and measures.

Table 1. Study Outcomes and Measures

Outcome Domain	Outcome	Measures	Baseline (1 Week Prior to Intervention)	Post Intervention (1 Month Post Intervention)	Follow-Up (8 Months Post Intervention)
**Child aggression – combined measure (primary outcome)**	Child aggression - combined measure	Combined score (mean of z-scores) of:<br>Child Behaviour Checklist – aggression subscale<br>ELDEQ study questionnaire aggression score	✓<br>p1zagg		✓<br>p3zagg
**Child aggression – individual measures (secondary outcomes)**	Child aggression - individual measures	Child Behaviour Checklist – aggression subscale	✓<br>p1cbclfinal		✓<br>p3cbclagress
		ELDEQ study questionnaire aggression score	✓<br>p1eldeqfinal		✓<br>p3eldeqfinal
		Filmed LabTab – aggression score	✓<br>p1labtab_manger		✓<br>p3labtab_manger
		Combined score (mean of z-scores) of:<br>Filmed 'Don't touch' – child behaviour<br>Filmed 'Clean Up' – child behaviour	✓<br>p1zcbehav		✓<br>p3zcbehav
		Interviewer rating – child defiance	✓<br>p1negativ		✓<br>p3negativ
**Child development (secondary outcomes)**	Child language	Combined score (mean of z-scores) of:<br>Expressive language task<br>Receptive language task	✓<br>p1childlang		✓<br>p3childlang
		Expressive language task	✓<br>p1tvesum		✓<br>p3tvesum
		Receptive language task	✓<br>p1tvasum		✓<br>p3tvasum
	Child attention and executive functions	Strengths and Difficulties Questionnaire – attention/hyperactivity subscale	✓<br>p1sdqfinal		✓<br>p3sdqhiperac
		Filmed Play Alone task – focus score	✓<br>p1playalone_mquality		✓<br>p3playalone_mquality
		Interviewer rating – child attention	✓<br>p1childatt		✓<br>p3childatt
		Go no Go task from the Early Years Toolbox	✓<br>p1cgng		✓<br>p3cgng
		Block Design task	✓<br>p1cblock		✓<br>p3block

Outcome Domain	Outcome	Measures	Baseline<br>(1 Week Prior to Intervention)	Post Intervention<br>(1 Month Post Intervention)	Follow-Up<br>(8 Months Post Intervention)
		Card Sort Task from the Early Years Toolbox	*Note: not measured at baseline*		✓<br>p3cs_switchacc
	Child empathy-prosocial behaviour	Combined score (mean of z-scores) of:<br>Em-Que questionnaire – Emotion Contagion<br>Strengths and Difficulties Questionnaire - Prosocial behaviour score	✓<br>p1zempat		✓<br>p3zempat
		Em-Que questionnaire – Emotion Contagion	✓<br>p1emqueemcont		✓<br>p3emqueemcont
		Strengths and Difficulties Questionnaire - Prosocial behaviour score	✓<br>p1sdqprosocial		✓<br>p3sdqprosocial
		Filmed Help Task	✓<br>p1helpt_score		✓<br>p3helpt_score
		Denham's puppet task	✓<br>p1puppetfinal		✓<br>p3puppetfinal
		Dictator Game	*Note: not measured at baseline*		✓<br>p3altruism2
	Theory of Mind	Triangle task	✓<br>p1ctrianaction		
		Sally-Anne task	*Note: not measured at baseline*		✓<br>p3sallyfinal
**Parenting (secondary outcomes)**	Positive parenting	Combined score (mean of z-scores) of:<br>Filmed Responsive Interactions – sensitivity<br>Filmed book-sharing - sensitivity<br>Filmed book-sharing task – reciprocity<br>Filmed Don't touch – Guidance<br>Filmed Clean Up – Guidance	✓<br>p1zpparent	✓<br>p2zpparent	✓<br>p3zpparent
		PAFAS - positive encouragement subscale	✓<br>p1pafasposen	✓<br>p2pafasposen	✓<br>p3pafasposen
		PAFAS - parent-child relationship subscale	✓<br>p1pafaspcrelat	✓<br>p2pafaspcrelat	✓<br>p3pafaspcrelat
		Filmed Book-sharing task - sensitivity	✓<br>p1bssensitivity	✓<br>p2bssensitivity	✓<br>p3bssensitivity
		Filmed Book-sharing task – reciprocity	✓<br>p1bsreciprocity	✓<br>p2bsreciprocity	✓<br>p3bsreciprocity
		Filmed Responsive Interactions task - sensitivity	✓<br>p1respint_total	✓<br>p2respint_total	✓<br>p3respint_total

Outcome Domain	Outcome	Measures	Baseline (1 Week Prior to Intervention)	Post Intervention (1 Month Post Intervention)	Follow-Up (8 Months Post Intervention)
		Filmed Don't Touch and Clean up tasks - mean of total z-scores guidance on two tasks	✓ p1ztotguid	✓ p2ztotguid	✓ p3ztotguid
	Harsh parenting	PAFAS questionnaire - coercive subscale	✓ p1pafascoer	✓ p2pafascoer	✓ p3pafascoer
		Filmed 'Don't touch' and 'Clean Up' Tasks – mean of coercion total z-scores on two tasks	✓ p1zcoercion	✓ p2zcoercion	✓ p3zcoercion
	Attitudes about punishment	Attitudes about physical punishment	✓ p1aappfinal	✓ p2aappfinal	
	Maltreatment	Juvenile Victimization Questionnaire - maltreatment	✓ p1jvqanymalt		✓ p3jvqanymalt
**Stress (secondary outcomes)**	Maternal stress	Perceived Stress Scale	✓ p1pssfinal	✓ p2pssfinal	
		Pelotas Parenting Stress Index	✓ p1psifinal	✓ p2psifinal	
	Maternal cortisol	3-month cortisol levels from hair samples	✓ p1mhairconc		✓ p3mhairconc
	Child cortisol	3-month cortisol levels from hair samples	✓ p1chairconc		✓ p3chairconc
* Measures applied pre-baseline in context of 24-month assessment of birth cohort study in which the trial is nested.					

The following additional data were collected prior to baseline, as part of cohort assessments:

- Maternal education measured in the perinatal assessment, and complemented with pre-natal data when perinatal was missing [abmateduc]
- Socioeconomic status measured when children were 24 months of age [fw24income]
- Frequency of reading-storytelling to child when children were 24 months of age [fw24reading]
- Child language when children were 24 months (to be used for missing language data at baseline)
    - Total language score: [fw24meanlanguage]
    - Expressive language score [fw24meanexpressilanguage]
    - Receptive language score [fw24meanreceplanguage]

The following additional data were collected at baseline:

- Child age [p1cage, p1cagecat]
- Child sex [p1sex]
- Neighbourhood [p1area]
- Maternal ethnicity [p1mrace]
- Mother relationship status [p1partner]
- Time mother spends with child per week [p1stayweek]
- Child attendance at preschool [p1school]
- Child callous unemotional traits [p1icufinal]
- Intimate partner violence [p1vpifinalcat]
- Maternal depression [p1epdsfinal]
- Maternal problem drinking [p1auditfinalcat]
- Hair cortisol concentration confounders variables for children [p1cusecort, abmateduc, fw24cbmi, p1jvqmalt]
- Hair cortisol concentration confounders variables for mothers [p1musecort, abmateduc, fw24matimc]

The following additional data were also collected about intervention adherence:

- Mother completed the intervention (yes/no) [p3attend]
  This was defined as attended 7+ out of 9 of the ACT intervention sessions and 6+ out of the 8 book sharing sessions in the DBS intervention group

The following additional data were also collected at post-intervention:

- Child language (combined receptive and expressive score) [p2zmeanlang], which will be analysed as a possible mediator of effects on the primary outcome.
- Child attention-hyperactivity (SDQ score) [p2sdqhiperac], which will be analysed as a possible mediator of effects on the primary outcome.

During the intervention phase, the number of sessions attended was recorded, and carer report of compliance and assessment of intervention quality and usefulness is also recorded at post treatment. Fidelity of facilitator implementation of the two parent-training programmes is also assessed by the research supervisors of each intervention.

Statistical principles

Significance level

All applicable statistical tests will be two-sided and will be performed using a 5% significance level. All confidence intervals presented will be 95% and two-sided.

Multiple comparisons

No formal adjustment for multiple testing will be made for the primary endpoints. The endpoints are associated with each other and an adjustment would over-correct (Schulz et al 2005).

All primary outcomes will be reported and interpreted together as specified in the primary hypothesis.

No formal correction will be made for multiple testing in the secondary and tertiary/exploratory analyses, but account will need to be taken in the interpretation where multiple statistical tests have been performed.

Interpretation of results will also take account of consistency across outcomes as well as clinical plausibility based on prior knowledge.

Analysis Populations

The intention- to- treat (ITT) population will be used for all analyses. This population includes all participants who were enrolled and randomised. Subjects will be analysed based on the groups to which they were randomized.

The per-protocol population will be used for a sensitivity analysis on the primary endpoints and key secondary outcomes. This population is a subset of the ITT population which excludes subjects who were allocated to but did not complete the ACT and DBS interventions. All ITT subjects in the control group will be included.

If the per-protocol population contains more than 95% of the ITT population then the sensitivity analysis will not be conducted.

Outliers

It is planned that all data will be included in the relevant analyses. However, if it is deemed necessary any subjects excluded from the summaries and/or statistical analyses will be documented along with the reason for exclusion in the report.

Trial population

The number of subjects who were enrolled, randomized and completed will be summarised and listed by intervention group. The number of subjects included in each population will also be presented by intervention group.

The consort diagram comprising the number of people screened/approached, eligible, randomised, received their allocated intervention, withdrawing/lost to the follow-up, will be produced in collaboration with the trial manager.

A listing of subjects removed from any population will be produced. Reports of compliance/attendance will also be listed and summarised.

Efficacy Analysis

General analysis considerations

For all analyses, the relevant assumptions will be checked. Alternative models may be used if necessary. If the normality assumption does not hold then a transformation such as log transformation, or an alternative distribution will be investigated. If there is no suitable transformation then a non-parametric testing method will be utilised.

Adjusted means and intervention group differences along with 95% confidence intervals and standard errors will be presented in a table, p-values will be presented for the difference between groups at post-intervention (where available) and follow-up. The primary comparisons between intervention and control group in child outcomes will be at follow-up.

Descriptive statistics

The primary endpoints, as well as the individual components, will be listed, and summarised by intervention group, and, where available, timepoint.

Demographic and baseline characteristics will be listed and summarised by intervention group. Categorical data will be summarised by numbers and percentages. Continuous data will be summarised by mean, SD, median, minimum and maximum values. The number and percent of missing data per outcome, group and time point will be summarised.

Missing data

For composite endpoints, missing data will be imputed on the individual measures and the composite will then be derived from the imputed components. If more than 1/3 of items on a questionnaire or measure are missing then the result will be considered missing for that person for that timepoint, otherwise prorated scores will be calculated and considered non-missing.

Due to technical difficulties language measures at baseline are missing for about one third of the participants. For the main analyses, multiple imputation will be used for these missing data, using previous measures of language measured at 24 months (pretrial cohort measure). In sensitivity analyses, this pretrial language measure will be used as a proxy for baseline language data for the whole cohort.

Primary analyses

Intervention effects will be assessed at post intervention and follow-up, and will be adjusted for: baseline scores, child age and sex, maternal education and depression, neighbourhood (as random effect), and additional, outcome-specific covariates shown in Table 2.

A mixed effects model will be fitted to the outcomes at post-intervention and at follow-up. Intervention, timepoint, intervention by timepoint interaction, covariates as listed above, and baseline values, will be fitted as fixed effects, neighbourhood and subject will be fitted as random effects, with repeated measures within a subject being accounted for. If the necessary assumptions of the models do not hold, suitable alternative models will be explored. Intention-to-treat analysis will be used to examine intervention effects.

Sensitivity analyses

Sensitivity analyses of the primary and main secondary endpoints will be conducted using the per-protocol population.

Table 2. Study Covariates for each Outcome

Outcome Domain	Outcome	Generic Study Covariates	Additional Covariates<br>(measured at baseline, or pre-baseline from cohort)
**Child aggression, combined measure (primary outcome)**	Child aggression	Baseline measure<br>Child age<br>Child sex<br>Maternal education<br>Maternal depression<br>Neighbourhood	Child maltreatment<br>PAFAS coercive parenting<br>Combined child language<br>Child Go no Go Task<br>Child callous-unemotional traits
**Child development (secondary outcomes)**	Child language	Baseline measure<br>Child age<br>Child sex<br>Maternal education<br>Maternal depression<br>Neighbourhood	Child maltreatment<br>Child preschool attendance<br>Reading-storytelling at 24m<br>Combined positive parenting
	Child attention and executive functions	Baseline measure (Card-Sort proxy = language)<br>Child age<br>Child sex<br>Maternal education<br>Maternal depression<br>Neighbourhood	Child maltreatment<br>PAFAS coercive parenting<br>Combined positive parenting
	Child empathy-prosocial behaviour	Baseline measure (Puppets proxy = language)<br>Child age<br>Child sex<br>Maternal education<br>Maternal depression<br>Neighbourhood	Child maltreatment<br>PAFAS coercive parenting<br>Combined positive parenting
	Child Theory of Mind	Baseline measure (Sally-Anne proxy = Triangle Task)<br>Child age<br>Child sex<br>Maternal education<br>Maternal depression<br>Neighbourhood	Child maltreatment<br>PAFAS coercive parenting<br>Combined positive parenting<br>Denham's puppet task<br>Child language<br>Older age sibling

Outcome Domain	Outcome	Generic Study Covariates	Additional Covariates<br>(measured at baseline, or pre-baseline from cohort)
	Positive parenting	Baseline measure<br>Child age<br>Child sex<br>Maternal education<br>Maternal depression<br>Neighbourhood	Maternal problem drinking<br>Reading-storytelling 24m [fw24reading]<br>Combined child aggression<br>Intimate partner violence<br>Pelotas Parenting Stress Index
**Parenting (secondary outcomes)**	Harsh parenting	Baseline measure<br>Child age<br>Child sex<br>Maternal education<br>Maternal depression<br>Neighbourhood	Maternal problem drinking<br>Combined child aggression<br>Intimate partner violence<br>Pelotas Parenting Stress Index<br>Attitudes about physical punishment<br>JVQ maltreatment
	Parental attitudes about corporal punishment	Baseline measure<br>Child age<br>Child sex<br>Maternal education<br>Maternal depression<br>Neighbourhood	Combined child aggression<br>Intimate partner violence<br>PAFAS coercive parenting
	Maltreatment	Baseline measure<br>Child age<br>Child sex<br>Maternal education<br>Maternal depression<br>Neighbourhood	Maternal problem drinking<br>Combined child aggression<br>Intimate partner violence<br>Pelotas Parenting Stress Index<br>Attitudes about physical punishment<br>PAFAS coercive parenting
	Maternal stress	Baseline measure<br>Child age<br>Child sex<br>Maternal education<br>Maternal depression<br>Neighbourhood	Intimate partner violence<br>Combined child aggression
**Stress (secondary outcomes)**	Maternal cortisol	Baseline measure<br>Maternal education<br>Maternal depression<br>Neighbourhood	Intimate partner violence<br>Combined child aggression<br>Maternal steroids use<br>Maternal BMI

Outcome Domain	Outcome	Generic Study Covariates	Additional Covariates (measured at baseline, or pre-baseline from cohort)
	Child cortisol	Baseline measure<br>Child age<br>Child sex<br>Maternal education<br>Maternal depression<br>Neighbourhood	PAFAS coercive parenting<br>Child maltreatment<br>Child steroids use<br>Child BMI<br>Intimate partner violence

Mediator effects

To test the role of possible mediators of the intervention effects on child outcomes, we will use a multiple multilevel Mediator Model fitted using the ml_mediation package in Stata or similar. Table 3 shows the mediators that will be tested for each outcome.

For each outcome, a single outcome measure will be selected for the mediation analyses, contingent on the results of the primary analyses. Table 3 shows the principal outcome measure that will be considered for mediator (and moderator) analyses for each outcome. If effects are found ($p<.05$) in the primary analyses for the principal outcome measure shown in Table 3, then mediation analyses will be run on that principal outcome measure. However, if no main effect is found for the principal outcome measure, then the mediation analyses will be run on whichever other measure of that outcome has the largest significant effect.

Separate models will be fitted for each outcome and follow the main analysis modelling strategy. Mediators will be tested for both interventions, unless specified otherwise in Table 3.

Moderator effects

Table 3 shows the potential moderators of intervention impact that will be tested for each outcome. To assess the effect of potential moderators, the same model will be fitted as described in 6.2 with the addition of the moderator of interest and moderator*intervention interaction. Moderators will be tested for both interventions, unless specified otherwise in Table 3.

Table 3. Mediators and Moderators for each Outcome

Outcome Domain	Outcome	Principal Outcome Measure for Moderator-Mediator Analyses	Moderators<br>(measured baseline, or pre-baseline from cohort)	Mediators<br>(post intervention unless stated otherwise)
**Child aggression, combined measure (primary outcome)**	Child aggression - combined measure	**Combined (mean) of z-scores from:**<br>Child Behaviour Checklist – aggression subscale<br>ELDEQ study questionnaire aggression score	Baseline measure of outcome<br>Child sex<br>Child age<br>Child callous-unemotional traits<br>Maternal education<br>Maternal depression<br>Intimate partner violence<br>PAFAS coercive parenting<br>Participation in PIM programme	PAFAS coercive parenting<br>Combined positive parenting<br>Combined child language (DBS only)<br>Child SDQ attention/ hyperactivity (DBS only)<br>Child cortisol at follow-up
**Child development (secondary outcomes)**	Child language (combined expressive and receptive)	**Combined (mean) of z-scores from:**<br>Teste de Vocabulário Expressivo<br>Teste de Vocabulário Receptivo	Baseline measure of outcome<br>Child sex<br>Child age<br>Maternal education<br>Maternal depression<br>Intimate partner violence<br>Filmed book-sharing task – sensitivity (DBS only)	Filmed book-sharing task – sensitivity (DBS only)<br>Filmed book-sharing task – reciprocity (DBS only)
	Child attention	**Strengths and Difficulties Questionnaire attention/hyperactivity subscale**	Baseline measure of outcome<br>Child sex<br>Child age<br>Maternal education<br>Maternal depression<br>Intimate partner violence<br>Filmed book-sharing task – sensitivity (DBS only)	Filmed book-sharing task – sensitivity (DBS only)<br>Filmed book-sharing task – reciprocity (DBS only)
	Child executive functions	**Block design score**	Baseline measure of outcome<br>Child sex<br>Child age<br>Maternal education<br>Maternal depression<br>Intimate partner violence	Combined average score of positive parenting

Outcome Domain	Outcome	Principal Outcome Measure for Moderator-Mediator Analyses	Moderators (measured baseline, or pre-baseline from cohort)	Mediators (post intervention unless stated otherwise)
	Child empathy-prosocial behaviour	**Combined (mean) of z-scores from:**<br>Em-Que questionnaire Emotion Contagion<br>Strengths and Difficulties Questionnaire - Prosocial behaviour score	Baseline measure of outcome<br>Child sex<br>Child age<br>Maternal education<br>Maternal depression<br>Intimate partner violence	Combined average score of positive parenting
	Child Theory of Mind	**Triangle task/ Sally-Anne task**	*None to be tested*	*None to be tested*
	Positive parenting	**Combined (mean) of z-scores from:**<br>Filmed Responsive Interactions – sensitivity<br>Filmed book-sharing - sensitivity<br>Filmed book-sharing task – reciprocity<br>Filmed Don't touch - Guidance<br>Filmed Clean Up – Guidance	Baseline measure of outcome<br>Child sex<br>Child age<br>Maternal education<br>Maternal depression	*None to be tested*
	Harsh parenting	**PAFAS questionnaire - coercive subscale**	Baseline measure of outcome (ACT only)<br>Child sex (ACT only)<br>Child age (ACT only)<br>Maternal education (ACT only)<br>Maternal depression (ACT only)<br>Intimate partner violence (ACT only)	Attitudes about physical punishment (ACT only)<br>Pelotas Parenting Stress Index (ACT only)
	Maltreatment	**Juvenile Victimisation Questionnaire (JVQ) – Maltreatment**	Baseline measure of outcome (ACT only)<br>Child sex (ACT only)<br>Child age (ACT only)<br>Maternal education (ACT only)<br>Maternal mental health (ACT only)<br>Intimate partner violence (ACT only)	Attitudes about physical punishment (ACT only)<br>Pelotas Parenting Stress Index (ACT only)
	Parental Attitudes about corporal punishment	**Attitudes about physical punishment**	*None to be tested*	*None to be tested*
**Stress (secondary outcomes)**	Maternal Stress	**Perceived Stress Scale/ Pelotas Parenting Stress Index**	*None to be tested*	*None to be tested*
	Maternal cortisol	**3-month cortisol levels from hair samples**	Baseline measure of outcome<br>Maternal education<br>Maternal depression<br>Intimate partner violence	Pelotas Parenting Stress Index

Outcome Domain	Outcome	Principal Outcome Measure for Moderator-Mediator Analyses	Moderators (measured baseline, or pre-baseline from cohort)	Mediators (post intervention unless stated otherwise)
	Child cortisol	**3-month cortisol levels from hair samples**	Baseline measure of outcome<br>Child age<br>Child sex<br>Maternal education<br>Maternal depression<br>Intimate partner violence	Pelotas Parenting Stress Index<br>PAFAS coercive parenting<br>Combined positive parenting

Changes from protocol defined statistical analysis

Small corrections, adjustments and clarifications to the trial protocol have been made in this analysis plan.

First, the protocol specified that the primary outcome of the trial was child aggression, but it did not specify if all measures of child aggression would be reported as primary outcomes. The analysis plan defines one combined measure of child aggression as the primary outcome, and the individual measures of child aggression as secondary outcomes. (see Table 1).

Second, in some parts of the protocol text, some of the secondary outcomes were referred to as "main secondary outcomes and others as "additional secondary outcomes". In the analysis plan all outcomes that are not "primary" are treated as secondary (see Table 1).

Third, some of the individual measures in the protocol have been changed:

1. For child attention, the Filmed Play Alone Task will be used as an additional measure and analysed as defined in this analysis plan (see Table 1).
2. The Card Sort Task measure was wrongly shown as a measure of child attention in the protocol, and will be analysed as a measure of child executive function (see Table 1).
3. Interviewers made short assessments of child behaviour after completing assessments. In the protocol it was planned to include a measure of self-control from these ratings, but the items were considered to better reflect child attention and child aggression. So, the analysis plan includes no interviewer rating of executive function, and includes instead additional interviewer ratings of child attention and aggression (see Table 1).
4. For child prosocial behaviour (called empathy/theory of mind in the protocol), the Strengths and Difficulties Questionnaire prosocial subscale score had not been specified in the protocol, but will be used as an additional measure of prosocial behaviour in the analyses (see Table 1).
5. The protocol stated that child protection service records would be measured as one measure of child maltreatment, but these records were not collected (because rates of official maltreatment in the population were discovered to be too low for analysis in this study), and so child protection service records will not be analysed.

Fourth, in the protocol it was stated that positive parenting would be measured at post intervention, and harsh parenting would be measured at follow-up, and hence they would be analysed accordingly. During the trial, parenting assessments were at both time points, and both positive and harsh parenting outcomes will be analysed at post-intervention and at follow-up, as defined in this analysis plan (see Table 1).

Fifth, the protocol stated that missing data would be handled using multiple imputation. However, the mixed effects models specified will handle the missing data without the requirement for formal imputation.

References

Cohen J, Cohen P, West SG, Aiken LS (2003). Applied multiple regression and correlation for the behavioural sciences (3rd ed.). Mahwah, NJ: Lawrence Erlbaum Associates.

De Boer MR, Waterlander WE, Kuijper LDJ, Steenhaus IHM, Twisk JWR. Testing for baseline differences in randomized controlled trials: an unhealthy research behaviour that is hard to eradicate. International Journal of Behavioural Nutrition and Physical Activity. (2015)12:4.

Fenson L, Pethick S, Renda C, Cox JL, Dale PS, Reznick JS. Short form versions of the MacArthur Communicative Development Inventories. Applied Psycholinguistics. 2000;21(1):95–115.

Hayes (2013). Introduction to Mediation, Moderation, and Conditional Process Analysis: a regression-model approach.

Hedges LV. Effect sizes in cluster-randomized designs. Journal of Educational and Behavioral Statistics. 2007;32(4):341–70.

National income deprivation affecting children Index (IDACI) (2015). https://www.northamptonshireanalysis.co.uk/linked-data/id/indicator/IDACINationalDectile.

Schulz KF, Grimes DA. Multiplicity in randomised trials I: endpoints and treatments. Lancet. 2005;365: 1591–95.

Vally Z, Murray L, Tomlinson M, Cooper PJ. The impact of dialogic book-sharing on training on infant language and attention: a randomized controlled trial in a deprived South African community. Journal of Child Psychology and Psychiatry. 2015;56(8):865-73.

Dowdall N (2015). The effectiveness of shared picture book reading interventions on child language outcomes: A systematic review and meta-analysis. Master's Thesis, Oxford University.

Goldfeld S, Quach J, Nicholls R, Reilly S, Ukoumunne OC, Wake M. Four-year-old outcomes of a universal infant-toddler shared reading intervention: The Let's Read trial. Archives of Pediatrics & Adolescent Medicine. 2012;166(11):1045-52.
